# Supplementary material for: Fluorinated trehalose analogues for cell surface engineering and imaging of Mycobacterium tuberculosis
Source: Chem Sci. 2024 Aug 12;15(34):13966–75. doi: 10.1039/d4sc00721b (PMC11317875; doi:10.1039/d4sc00721b)
Supplement: SC-015-D4SC00721B-s001 [file SC-015-D4SC00721B-s001.pdf]

Supplementary Information (SI) for Chemical Science.
This journal is © The Royal Society of Chemistry 2024

Fluorinated trehalose analogues for cell surface engineering and imaging of *Mycobacterium tuberculosis*

Collette S. Guy[a], James A. Gott[b], Jonathan Ramírez-Cárdenas[c], Christopher de Wolf[a], Christopher M. Furze[a], Geoff West[b], Juan C. Muñoz-García[c], Jesus Angulo[c], Elizabeth Fullam[a]*

[a] School of Life Sciences, University of Warwick, Coventry, CV4 7AL, UK
[b] WMG, University of Warwick, Coventry, CV4 7AL, UK
[c] Instituto de Investigaciones Químicas (IIQ), Consejo Superior de Investigaciones Científicas and Universidad de Sevilla, Avenida Américo Vespucio, 49, Sevilla, 41092, Spain.

*To whom correspondence should be addressed:

Elizabeth Fullam, School of Life Sciences, University of Warwick, Coventry, CV4 7AL, United Kingdom.
e.fullam@warwick.ac.uk; Tel. +44 (0)2476 574239

Table of Contents

Supplementary Results **4**
Scheme S1. Synthetic route for 4-deoxy-4-fluoro-trehalose 5
Fig. S1: Binding affinities for LpqY 6
Fig. S2. STD NMR study of the binding of LpqY with 2F-Tre, 4F-Tre and 6F-Tre with protein irradiation in the aliphatics spectral region 7
Fig. S3. STD NMR for LpqY with 2F-Tre, 4F-Tre and 6F-Tre with protein irradiation in the aromatics spectral region 8
Table S1. STD NMR data for the binding of LpqY with 2F-Tre 9
Table S2. STD NMR data for the binding of LpqY with 3F-Tre 10
Table S3. STD NMR data for the binding of LpqY with 4F-Tre 11
Table S4. STD NMR data for the binding of LpqY with 6F-Tre 12
Fig. S4. Differential Epitope Mapping by STD NMR of LpqY with 2F-Tre, 3F-Tre, 4F-Tre and 6F-Tre 13
Fig. S5. Molecular dynamic simulations of LpqY with 2F-Tre, 3F-Tre, 4F-Tre and 6F-Tre 14
Fig. S6. Comparison of the binding orientations of the F-Tre analogues 15
Fig. S7. Ion chromatography traces of F-Tre labelled *Mycobacterium tuberculosis* 16
Table S5. Retention times for ion chromatography traces of F-Tre labelled *Mycobacterium tuberculosis* 17
Table S6. *Mtb* cytosolic concentrations of F-Tre analogue uptake 18
Fig. S8. Ion chromatography traces of F-Tre labelled *Mycobacterium tuberculosis* 19
Table S7. Analysis of F-Tre uptake in *Mycobacterium tuberculosis* over time 20
Table S8. Analysis of F-Tre labelled *Mycobacterium bovis* BCG and LpqY-SugABC mutant strains 21
Fig. S9. Ion chromatography traces of hydrolysed lipid extracts F-Tre labelled *Mycobacterium tuberculosis* 22
Table S9. Analysis of hydrolysed lipid extracts F-Tre labelled *Mycobacterium tuberculosis* 23
Fig. S10. F-Glc metabolite analysis of *Mtb* labelled with F-Tre analogues 24
Table S10. Analysis of F-Glc metabolites 25
Fig. S11. SIMS mass spectra of *Mtb* labelled with F-Tre analogues
Experimental **26**
General Information and Procedures 26
^{1}H NMR, ^{13}C NMR and MS data 26
Bacterial strains, cell lines, culture conditions and chemicals 26
Expression and purification of TreT 26
Chemoenzymatic synthesis of fluorinated trehalose derivatives 27
2-deoxy-2-fluoro-trehalose (**2**) 27
3-deoxy-3-fluoro-trehalose (**3**) 27
6-deoxy-6-fluoro-trehalose (**5**) 28
Chemical synthesis of 4-deoxy-4-fluoro-trehalose 28
2,3,6,2′,3′,4′,6’,-hepta-*O*-benzoyl-α,α’-D-trehalose (**6**) 28
2,3,6,-tri-*O*-benzoyl-α-D-galactopyranosyl-(1→1)-2′,3′,4′,6′,-tetra-*O*-benzoyl-α-D-glucopyranoside (**8**) 28
4-fluoro-2,3,6,-tri-*O*-benzoyl-α-D-glucopyranosyl-(1→1)-2′,3′,4′,6′,-tetra-*O*-benzoyl-α-D-glucopyranoside (**9**) 29
4-deoxy-4-fluoro-trehalose (**4**) 30
Production and purification of LpqY 30
Microscale Thermophoresis 30
STD-NMR 31
Molecular dynamics 31
Input preparation and equilibration 31
MD simulation 32
Analysis of F-Tre uptake at a single-time point 32

Table S11: High performance anion exchange chromatography KOH elution gradient for F-Tre analysis 33
Time dependent F-Tre uptake 33
Analysis of F-Glc metabolites 34
Table S12: High performance anion exchange chromatography KOH elution gradient for F-Glc analysis 34
Lipid extraction and analysis 34
Preparation of samples for focussed ion beam (FIB) secondary ion mass spectrometry (SIMs) 35
Scanning electron microscopy (SEM) and FIB-SIMS 35
NMR Spectra **37**
Fig. S12. ^{1}H NMR 2-deoxy-2-fluoro-trehalose (**2**) 37
Fig. S13. ^{13}C NMR 2-deoxy-2-fluoro-trehalose (**2**) 37
Fig. S14. ^{19}F NMR 2-deoxy-2-fluoro-trehalose (**2**) 38
Fig. S15. ^{1}H NMR 3-deoxy-3-fluoro-trehalose (**3**) 38
Fig. S16. ^{13}C NMR 3-deoxy-3-fluoro-trehalose (**3**) 39
Fig. S17. ^{19}F NMR 3-deoxy-3-fluoro-trehalose (**3**) 39
Fig. S18. ^{1}H NMR of 4-deoxy-4-fluoro-trehalose (**4**) 40
Fig. S19. ^{13}C NMR of 4-deoxy-4-fluoro-trehalose (**4**) 40
Fig. S20. ^{19}F NMR of 4-deoxy-4-fluoro-trehalose (**4**) 41
Fig. S21. ^{1}H NMR of 6-deoxy-6-fluoro-trehalose (**5**) 41
Fig. S22. ^{13}C NMR of 6-deoxy-6-fluoro-trehalose (**5**) 42
Fig. S23. ^{19}F NMR of 6-deoxy-6-fluoro-trehalose (**5**) 42
Fig. S24. ^{1}H NMR of 2,3,6,2′,3′,4′,6’,-hepta-*O*-benzoyl-α,α’-D-trehalose (**6**) 43
Fig. S25. ^{13}C NMR of 2,3,6,2′,3′,4′,6’,-hepta-*O*-benzoyl-α,α’-D-trehalose (**6**) 43
Fig. S26. ^{1}H NMR of 2,3,6,-tri-*O*-benzoyl-α-D-galactopyranosyl-(1→1)-2′,3′,4′,6′,-tetra-*O*-benzoyl-α-D-glucopyranoside (**8**) 44
Fig. S27. ^{13}C NMR of 2,3,6,-tri-*O*-benzoyl-α-D-galactopyranosyl-(1→1)-2′,3′,4′,6′,-tetra-*O*-benzoyl-α-D-glucopyranoside (**8**) 44
Fig. S28. ^{1}H NMR of 4-fluoro-2,3,6,-tri-*O*-benzoyl-α-D-galactopyranosyl-(1→1)-2′,3′,4′,6′,-tetra-*O*-benzoyl-α-D-glucopyranoside (**9**) 45
Fig. 29. ^{13}C NMR of 4-fluoro-2,3,6,-tri-*O*-benzoyl-α-D-galactopyranosyl-(1→1)-2′,3′,4′,6′,-tetra-*O*-benzoyl-α-D-glucopyranoside (**9**) 45
Fig. S30. ^{19}F NMR of 4-fluoro-2,3,6,-tri-*O*-benzoyl-α-D-galactopyranosyl-(1→1)-2′,3′,4′,6′,-tetra-*O*-benzoyl-α-D-glucopyranoside (**9**) 46
References **47**

Scheme S1. Synthetic route for 4-deoxy-4-fluoro-trehalose. *Reagents and conditions:* a) 8.5 eq. benzoyl chloride, -40 °C, 2 h, room temperature, 48 h, b) 10 eq. pyridine, 2 eq. triflic anhydride, 0 °C, then room temperature, 3 h, c) 7 eq. sodium nitrite, DMF, room temperature, 22 h, d) 2.5 eq. diethylaminosulfur trifluoride (DAST), 40 °C, 72 h, e) 0.2 M methanolic NaOMe, room temperature, 14 h.

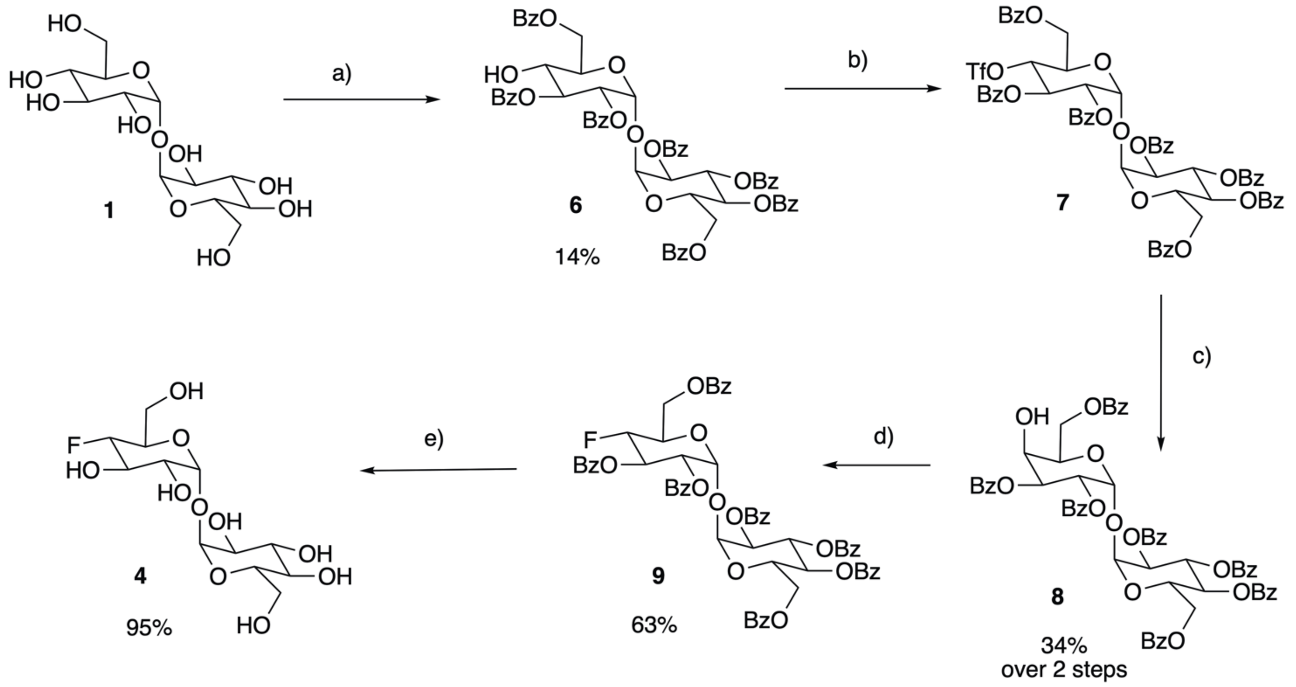

Fig. S1: Binding affinities for LpqY. Binding of trehalose, 2F-Tre, 3F-Tre, 4F-Tre and 6F-Tre to LpqY measured by microscale thermophoresis (MST). FNorm (%) is the normalized fluorescence signal of the change in MST signal. Error bars represent standard deviations from at least three independent experiments.

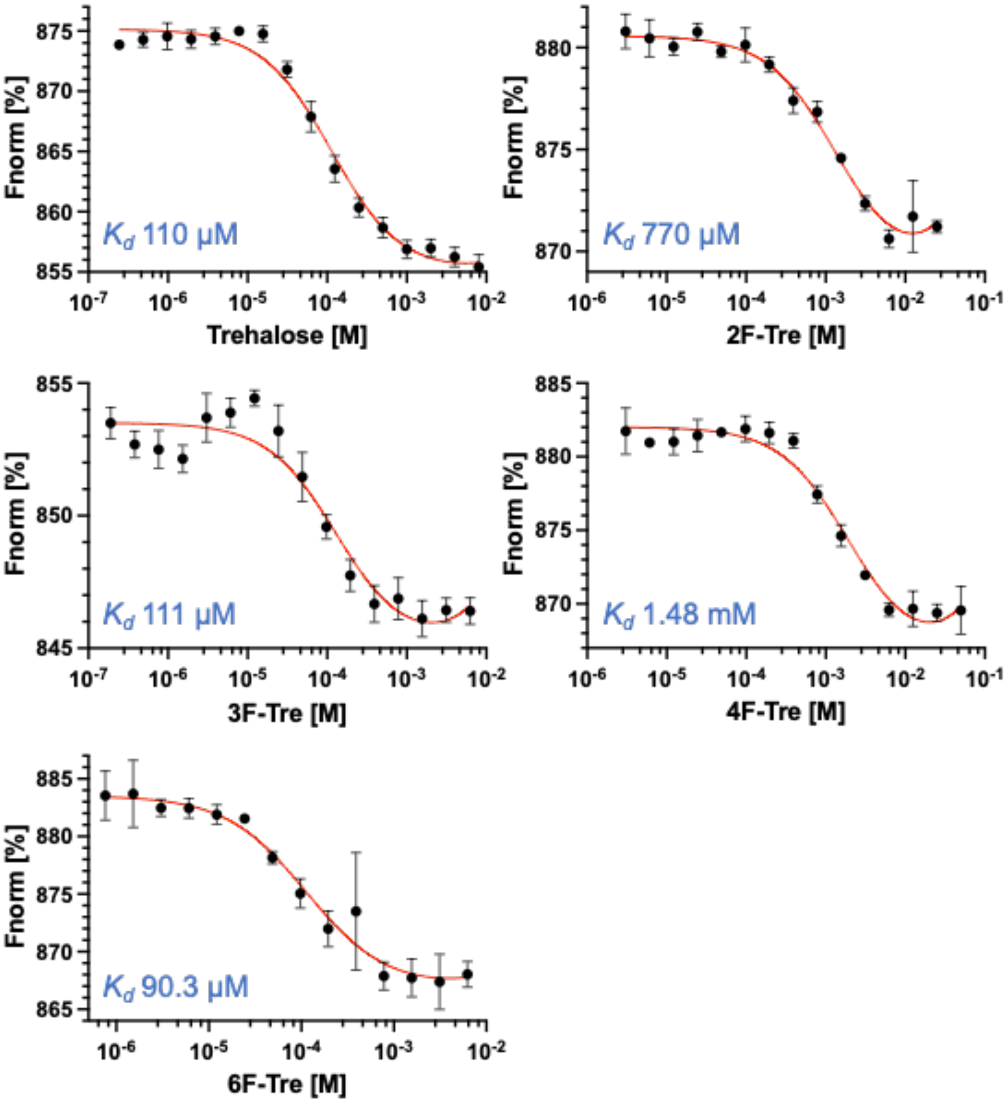

Fig. S2. STD NMR study of the binding of *Mtr* LpqY with 2F-Tre, 4F-Tre and 6F-Tre with protein irradiation in the aliphatics spectral region. STD NMR build-up curves for (A) 2F-Tre, (B) 4F-Tre and (C) 6F-Tre, in complex with *Mtr* LpqY. Temperature 5 °C for (**A**) and (**B**), and 30 °C for (**C**). Saturation frequency set at 0.53 ppm for (**A**) and (**B**), and 0.84 ppm for (**C**).

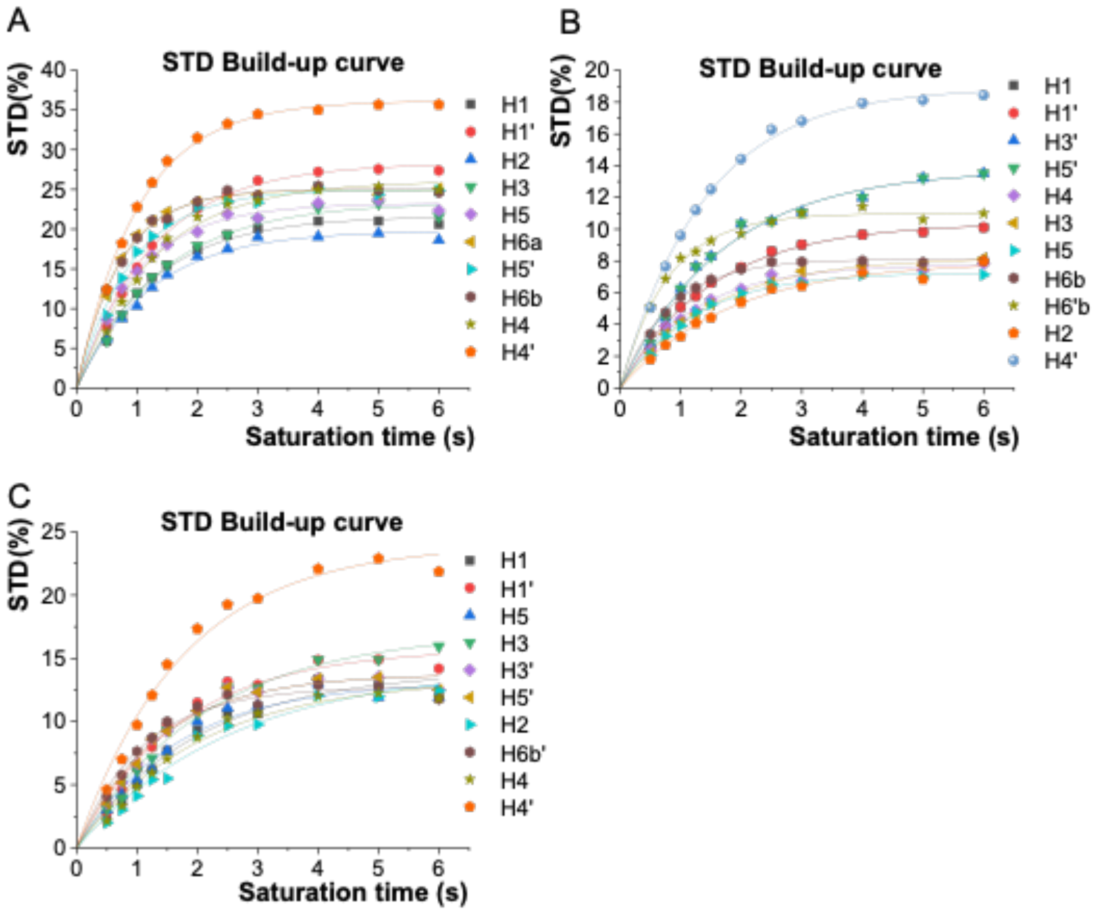

Fig. S3. STD NMR *for Mtr* LpqY with 2F-Tre, 4F-Tre and 6F-Tre with protein irradiation in the aromatics spectral region. STD NMR build-up curves for (A) 2F-Tre, (B) 4F-Tre and (C) 6F-Tre in complex with *Mtr* LpqY. Temperature 5 °C for (**A**) and (**B**), and 30 °C for (**C**). Saturation frequency set at 7.0 ppm for (**A**) and (**B**), and 7.24 ppm for (**C**).

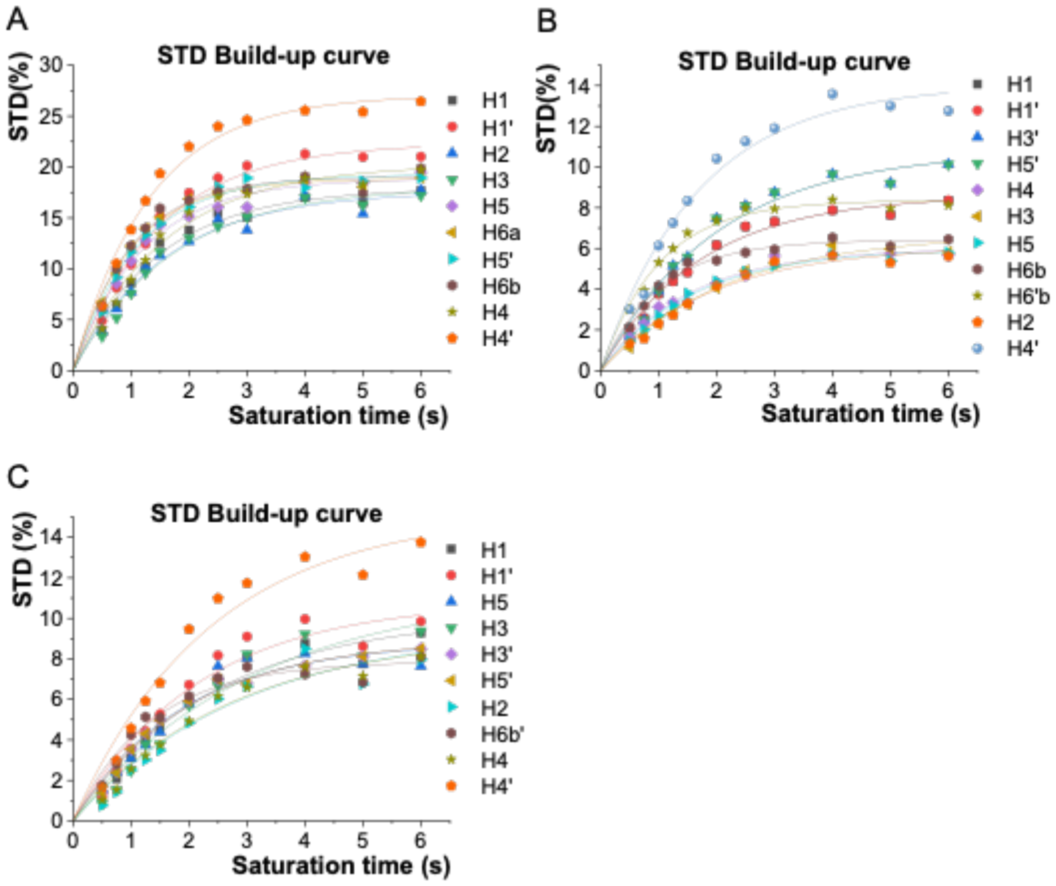

Table S1. STD NMR data for the binding of *Mtr* LpqY with 2F-Tre with protein irradiation in aliphatics (**A**) or in aromatics (**B**). Temperature 5 °C. Saturation frequency set at 0.53 ppm for (**A**) and at 7.00 ppm for (**B**).

A

	**k_{sat} (s^{-1})**	**STD^{max}**	**STD_0 (s^{-1})**	**STD_{rel} (%)**
**H1'**	0,79	28,30	22,29	63
**H4'**	0,98	36,18	35,27	100
**H5'**	1,10	24,99	27,57	78
**H6'**	--	n.d.	--	--
**H1**	0,80	21,63	17,23	49
**H2**	0,82	19,79	16,26	46
**H3**	0,71	23,36	16,62	47
**H4**	0,78	26,06	20,38	58
**H5**	1,00	23,22	23,14	66
**H6a**	1,41	24,93	35,10	100
**H6b**	1,38	25,05	34,50	98

B

	**k_{sat} (s^{-1})**	**STD^{max}**	**STD_0 (s^{-1})**	**STD_{rel} (%)**
**H1'**	0,68	22,31	15,21	75
**H4'**	0,75	27,08	20,28	100
**H5'**	0,91	19,25	17,42	86
**H6'**	--	n.d.	--	--
**H1**	0,68	17,89	12,22	60
**H2**	0,63	17,59	11,06	55
**H3**	0,59	18,03	10,63	52
**H4**	0,63	20,24	12,83	63
**H5**	0,83	18,89	15,67	77
**H6a**	1,02	18,81	19,19	95
**H6b**	1,02	19,03	19,40	96

Table S2. STD NMR data for the binding of *Mtr* LpqY with 3F-Tre at a single saturation time. STD factors and relative STDs obtained at a single saturation time (6 s) for 3-deoxy-3-fluoro-α,α′-trehalose in complex with *Mtr* LpqY with protein irradiation in aliphatics (**A**) or in aromatics (**B**). Temperature 30 °C. Saturation frequency set at 0.84 ppm for (**A**) and at 7.24 ppm for (**B**).

A

	**STD factor**	**STD_{rel} (%)**
**H1'**	2,79	79
**H2'**	2,79	79
**H4'**	3,55	100
**H6’**	n.d.	--
**H1**	2,48	70
**H2**	2,06	58
**H4**	1,53	43
**H5**	1,53	43
**H6b**	1,96	55

B

	**STD factor**	**STD_{rel} (%)**
**H1'**	1,66	79
**H2'**	1,78	85
**H4'**	2,10	100
**H6’**	n.d.	--
**H1**	1,58	75
**H2**	1,71	81
**H4**	1,37	65
**H5**	1,02	49
**H6b**	1,31	62

Table S3. STD NMR data for the binding of *Mtr* LpqY with 4F-Tre with protein irradiation in aliphatics (**A**) or in aromatics (**B**). Temperature 5 °C. Saturation frequency set at 0.53 ppm for (**A**) and at 7.00 ppm for (**B**).

A

	k_{sat} (s^{-1})	STD^{max}	STD_0 (s^{-1})	STD_{rel} (%)
**H1'/H1**	0,66	10,38	6,80	49
**H3'/H5'**	0,59	13,75	8,17	59
**H4'**	0,71	18,92	13,48	97
**H6'b**	1,27	10,99	13,92	100
**H2**	0,56	7,95	4,42	32
**H3**	0,67	8,20	5,49	39
**H4**	0,83	7,76	6,44	46
**H5**	0,82	7,29	5,95	43
**H6b**	1,20	8,11	9,72	70

B

	k_{sat} (s^{-1})	STD^{max}	STD_0 (s^{-1})	STD_{rel} (%)
**H1'/H1**	0,58	8,61	5,01	61
**H3'/H5'**	0,50	10,79	5,38	65
**H4'**	0,59	14,04	8,28	100
**H6'b**	0,99	8,36	8,28	100
**H2**	0,53	6,12	3,22	39
**H3**	0,46	6,72	3,09	37
**H4**	0,65	5,99	3,92	47
**H5**	0,63	5,97	3,76	45
**H6b**	0,99	6,40	6,36	77

Table S4. STD NMR data for the binding of *Mtr* LpqY with 6F-Tre with protein irradiation in aliphatics (**A**) or in aromatics (**B**). Temperature 30 °C. Saturation frequency set at 0.84 ppm for (**A**) and at 7.24 ppm for (**B**).

A

	k_{sat} (s^{-1})	STD^{max}	STD_0 (s^{-1})	STD_{rel} (%)
**H1'**	0,58	15,77	9,16	67
**H4'**	0,57	24,02	13,72	100
**H3'/H5'**	0,74	13,76	10,13	74
**H6'b**	0,92	12,72	11,69	85
**H1**	0,50	14,05	7,00	51
**H2**	0,39	14,32	5,59	41
**H3**	0,46	17,15	7,96	58
**H4**	0,49	13,39	6,58	48
**H5**	0,60	13,17	7,90	58

B

	k_{sat} (s^{-1})	STD^{max}	STD_0 (s^{-1})	STD_{rel} (%)
**H1'**	0,46	10,85	4,98	78
**H4'**	0,42	15,21	6,39	100
**H3'/H5'**	0,51	8,94	4,54	71
**H6'b**	0,73	7,89	5,79	91
**H1**	0,41	10,13	4,18	65
**H2**	0,34	9,50	3,27	51
**H3**	0,32	11,36	3,66	57
**H4**	0,36	9,30	3,37	53
**H5**	0,52	8,85	4,63	72

Fig. S4. Differential Epitope Mapping by STD NMR of *Mtr* LpqY with 2F-Tre, 3F-Tre, 4-F-Tre and 6F-Tre. Differential Epitope Mapping histograms with multifrequency irradiation (0.53 ppm/7.0 ppm) for **A**) 2F-Tre and **C**) 4F-Tre. A different multifrequency irradiation (0.84 ppm/7.24 ppm) was used for **B**) 3F-Tre (analysis carried out at a single saturation time of 6 s) and **D**) 6F-Tre in complex with *Mtr* LpqY. Positive DEEP-STD factors (ΔSTDs) indicate proximity towards aliphatic side chains in the binding site and are shown in orange, whereas negative ΔSTDs indicate proximity towards aromatic side chains in the binding site and are shown in blue. ΔSTD values were calculated as previously described.[1]

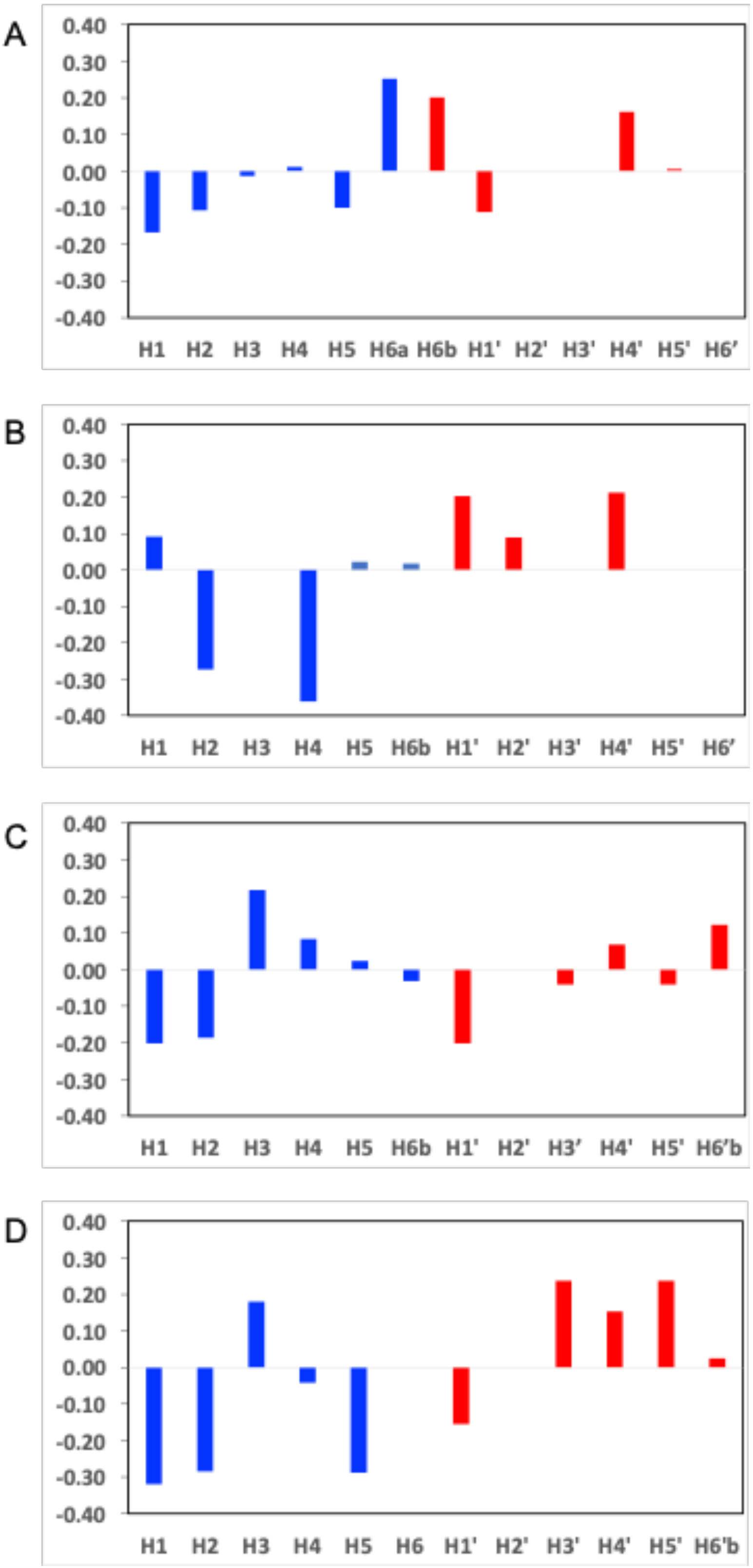

Fig. S5. Molecular dynamic simulations *Mtr* LpqY with 2F-Tre, 3F-Tre, 4F-Tre and 6F-Tre. Evolution of the ligand backbone RMSD (green) with respect to the protein binding site (*i.e.* 5 Å from the ligand) and evolution of the protein backbone RMSD (blue) over 100 ns of MD simulation of the complex between *Mtr* LpqY. (a) 2F-Tre, (b) 3-F-Tre, (c) 4F-Tre, and (d) 6-F-Tre

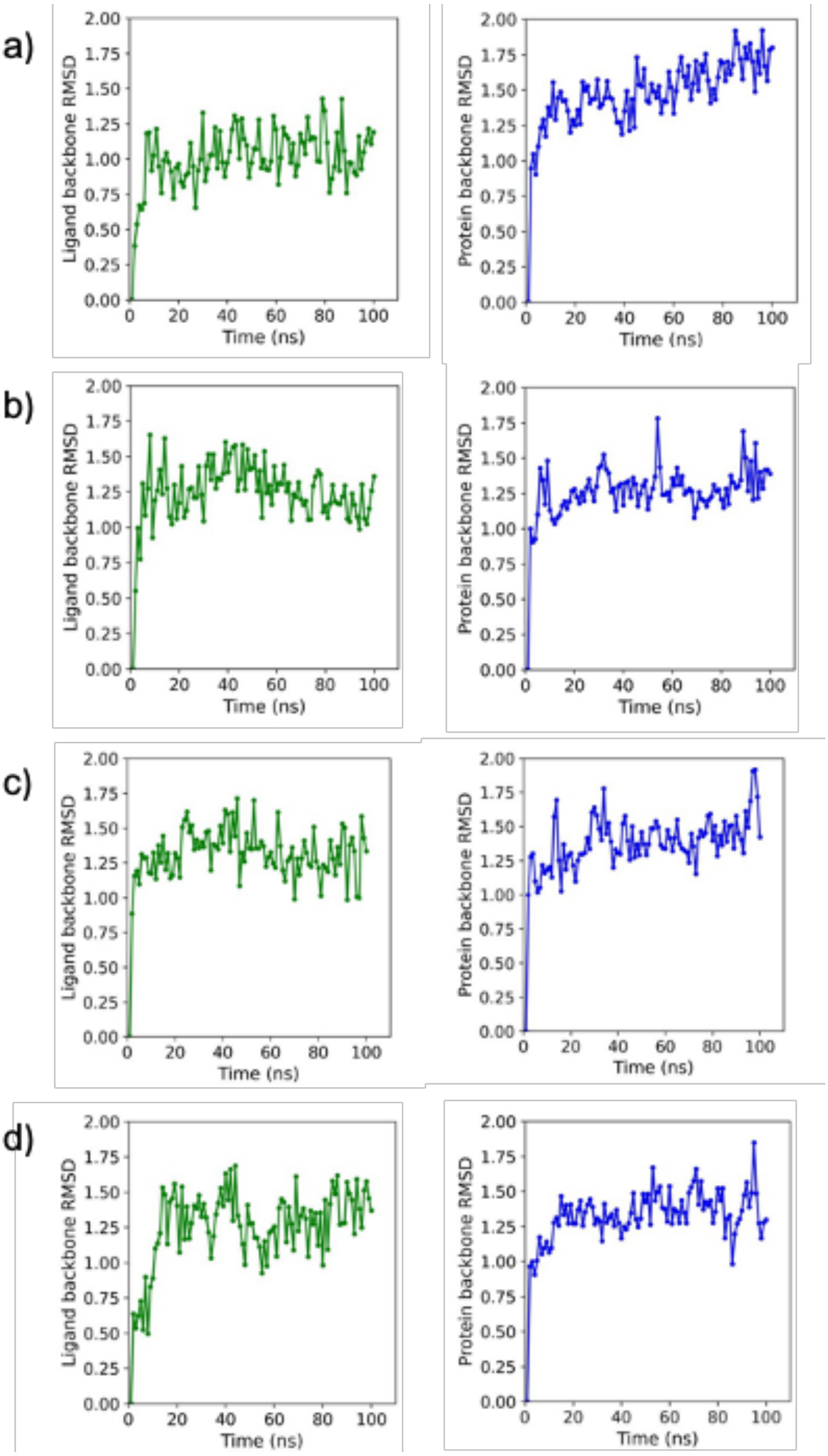

Fig. S6. **Comparison of the binding orientations of the F-Tre analogues**. Close up superposition showing the binding orientation of the trehalose ligand in stick representation (orange carbon atoms), (PDB 7APE), with the 100 ns molecular dynamic snapshots of 2F-Tre (blue carbon atoms), 3F-Tre (magenta carbon atoms), 4F-Tre (grey carbon atoms) and 6F-Tre (green carbon atoms). LpqY is shown in cartoon representation (white, PDB 7APE) and Arg404, which is at the base of the binding cavity, is highlighted. Oxygen, red; nitrogen, blue; fluorine, pale blue

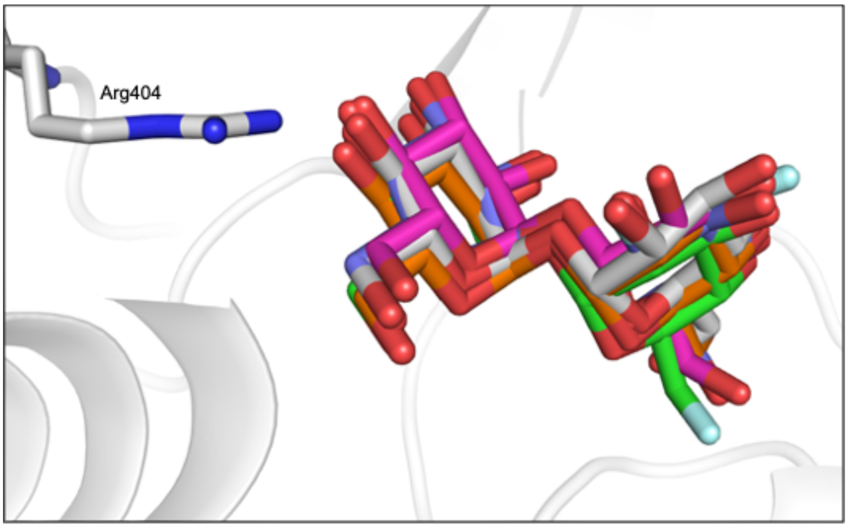

Fig. S7. Ion chromatography traces of F-Tre labelled *Mycobacterium tuberculosis.* *Mtb* was cultured in the presence of F-Tre analogues **2**-**5** (0 – 200 µM) and the cytosolic extracts analysed by high performance anion exchange chromatography with pulsed amperometric detection (HPAEC-PAD). The F-Tre cytosolic concentration was determined from the respective calibration curves for each F-Tre standard. The retention times and peak areas are in Table S5.

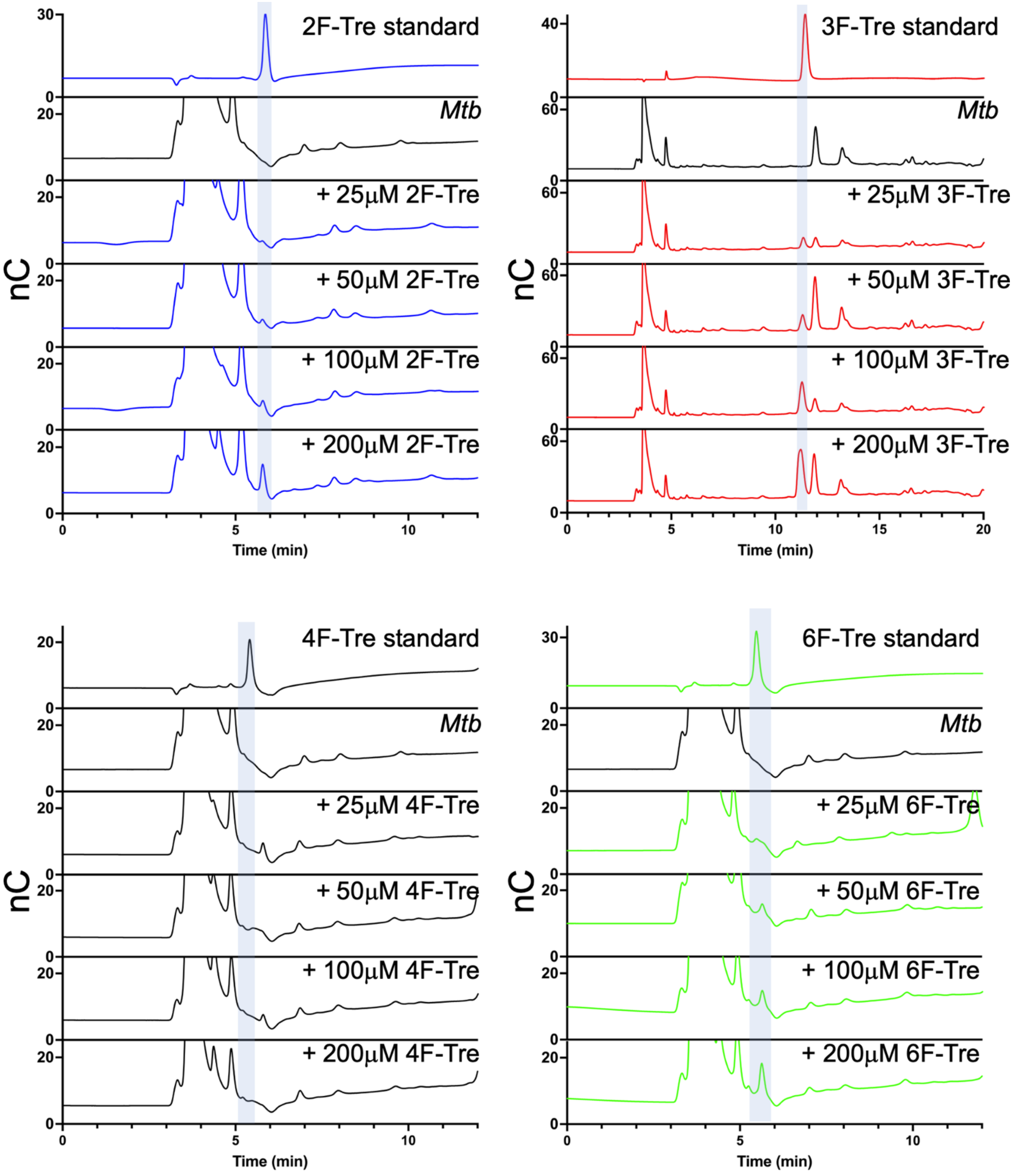

Table S5. Retention times for ion chromatography traces of F-Tre labelled *Mycobacterium tuberculosis*. Retention times and peaks areas of relevant peaks in HPAEC traces (Fig S7.) are shown. Peaks corresponding to F-Tre analogues are in bold.

	2F-Tre		3F-Tre		4F-Tre		6F_tre	
	Retention time (min)	Peak area (nC*min)	Retention time (min)	Peak area (nC*min)	Retention time (min)	Peak area (nC*min)	Retention time (min)	Peak area (nC*min)
F-Tre standard	**5.859**	**4.1741**	**11.417**	**9.721**	**5.400**	**2.818**	**5.467**	**4.546**
Mtb control	**-**	-	12.334	0.436	**-**	-	**-**	**-**
Mtb + 25 μM F-Tre	**5.767**	**0.096**	**11.325**<br>11.925	**1.869**<br>1.589	**-**	-	**5.467**	**0.144**
Mtb + 50 μM F-Tre	**5.775**	**0.206**	**11.309**<br>11.909	**3.093**<br>10.023	-	-	**5.642**	**0.506**
Mtb + 100 μM F-Tre	**5.784**	**0.362**	**11.267**<br>11.892	**6.779**<br>2.181	-	-	**5.634**	**0.866**
Mtb + 200 μM F-Tre	**5.784**	**1.328**	**11.225**<br>11.867	**12.530**<br>7.277	**-**	-	**5.625**	**1.604**

Table S6. ***Mtb*** **cytosolic concentrations of F-Tre analogue uptake from ion chromatography**

Time (h)	2F-Tre (2) (μM)	3F-Tre (3) (μM)	4F-Tre (4) (μM)	6F-Tre (5) (μM)
0	0 ± 0	0 ± 0	0	0 ± 0
0.5	636 ± 33	250 ± 102	0	342 ± 16
1	838 ± 52	423 ± 256	0	797 ± 224
1.5	1144 ± 250	1030 ± 335	0	1392 ± 83
2	1717 ± 151	1628 ± 25	0	1804 ± 111
4	2917 ± 105	3016 ± 102	0	2945 ± 234
6	4597 ± 236	5872 ± 297	0	4694 ± 237
8	6639 ± 337	7892 ± 72	0	6843 ± 43

Error bars denote the standard deviation from duplicate experiments.

Fig. S8. Ion chromatography traces of F-Tre labelled *Mycobacterium tuberculosis.* *Mtb* was cultured in the presence of F-Tre analogues **2-5** (100 µM) and the cytosolic extracts analysed at the time points (hour) indicated by high performance anion exchange chromatography with pulsed amperometric detection indicated (HPAEC-PAD). The F-Tre cytosolic concentration was determined from the calibration curves from each F-Tre standard. The retention times and peak areas are in Table S7.

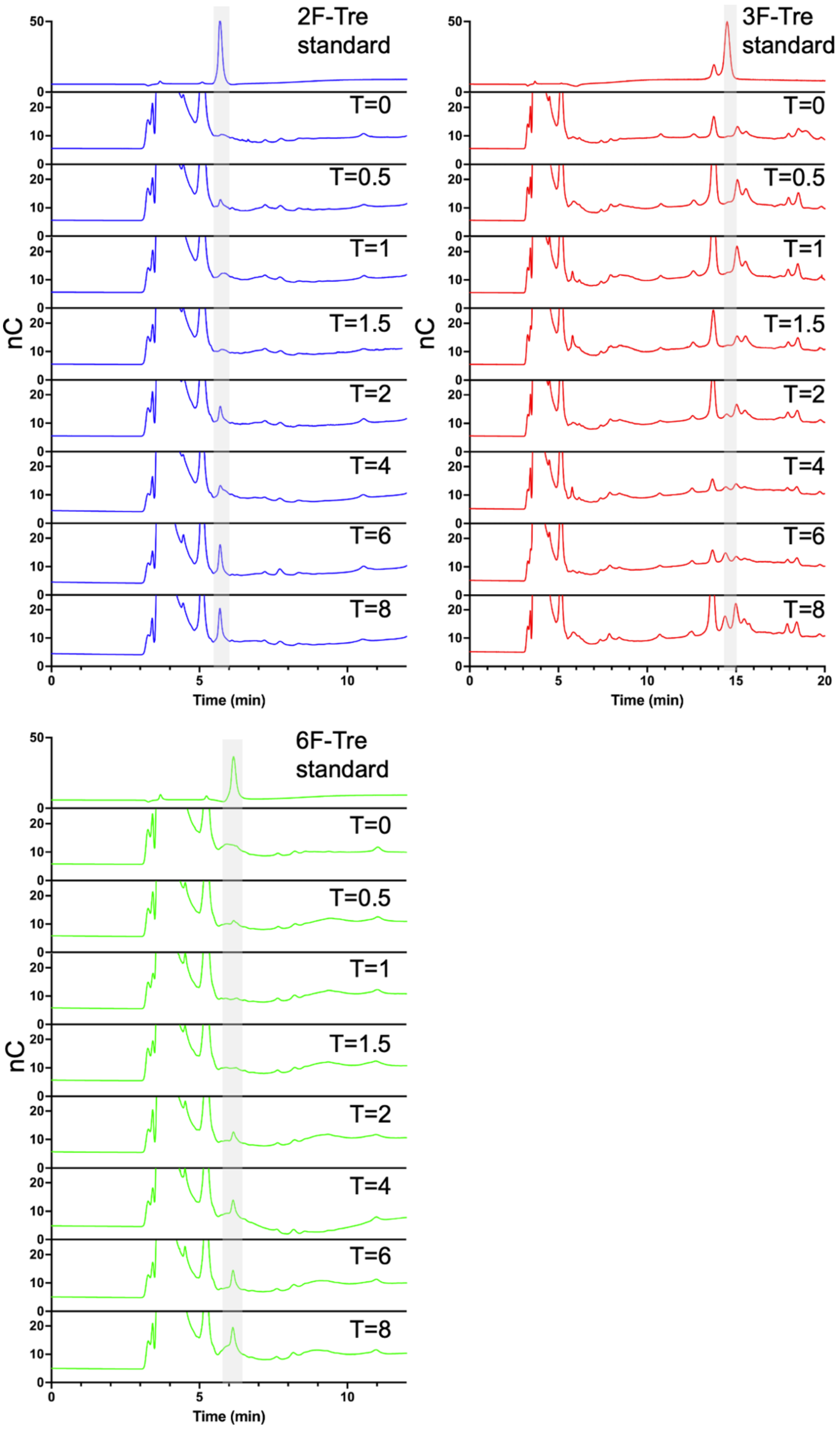

Table S7. Analysis of F-Tre uptake in *Mycobacterium tuberculosis* over time. Retention times and peaks areas of relevant peaks in HPAEC traces (Fig S8) are shown. Peaks corresponding to F-Tre analogue are in bold

	2F-Tre		3F-Tre		6F-Tre	
	Retention time (min)	Peak area (nC*min)	Retention time (min)	Peak area (nC*min)	Retention time (min)	Peak area (nC*min)
F-Tre Standard	**5.692**	**7.610**	**14.492**	**12.556**	**6.150**	**6.563**
T=0	-	-	13.759<br>15.075	1.915<br>0.686	-	-
T=0.5	**5.692**	**0.285**	13.742<br>**14.309**<br>15.059	8.289<br>**0.011**<br>0.667	**6.150**	**0.259**
T=1	**5.859**	**0.384**	13.734<br>**14.742**<br>15.050	7.926<br>**0.005**<br>0.727	**6.267**	**0.070**
T=1.5	**5.759**	**0.195**	13.717<br>**14.250**<br>15.042	2.959<br>**0.031**<br>0.466	**6.225**	**0.084**
T=2	**5.709**	**0.801**	13.709<br>**14.475**<br>15.034	4.426<br>**0.253**<br>0.821	**6.150**	**0.516**
T=4	**5.709**	**0.800**	13.684<br>**14.417**<br>15.017	0.364<br>**0.264**<br>0.347	**6.142**	**0.767**
T=6	**5.700**	**1.443**	13.675<br>**14.409**<br>15.009	0.902<br>**0.847**<br>0.333	**6.134**	**0.937**
T=8	**5.692**	**1.748**	13.659<br>**14.384**<br>14.984	4.666<br>**1.243**<br>1.631	**6.125**	**1.130**

Table S8. Analysis of F-Tre labelled *Mycobacterium bovis* BCG and LpqY-SugABC mutant strains. Retention times and peaks areas of relevant peaks in HPAEC traces (Fig. 7) are shown. Peaks corresponding to F-Tre analogue are in bold

	2F-Tre		3F-Tre		4F-Tre		6F-Tre	
	Retention time (min)	Peak area (nC*min)	Retention time (min)	Peak area (nC*min)	Retention time (min)	Peak area (nC*min)	Retention time (min)	Peak area (nC*min)
Tre standard	5.492	8.2887	5.492	8.2887	5.492	8.2887	5.492	8.2887
F-Tre standard	**6.367**	**21.393**	**18.117**	**5.999**	**6.508**	**10.769**	**6.625**	**10.946**
BCG + F-Tre	5.550<br>**6.392**	5.704<br>**1.506**	5.553<br>17.158<br>**18.225**<br>19.350	7.132<br>0.819<br>**1.071**<br>0.846	5.525	5.577	5.525<br>**6.642**	8.846<br>**1.122**
ΔLpqYSugABC + F-Tre	5.550	6.822	5.533<br>17.075<br>19.325	6.862<br>0.908<br>0.828	5.525	5.217	5.517	4.865
ΔLpqYSugABC: complemented + F-Tre	5.542<br>**6.375**	6.149<br>**0.581**	5.553<br>17.050<br>**18.108**<br>19.317	4.968<br>0.260<br>**0.285**<br>0.179	5.525	3.793	5.525<br>**6.633**	4.153<br>**0.279**

Fig. S9. Ion chromatography traces of hydrolysed lipid extracts F-Tre labelled *Mycobacterium tuberculosis.* To complement our TLC studies which identified new spots in F-Tre labelled *Mtb* cells, free sugars released from the lipid extracts of F-Tre (100 μM) labelled cells were analysed by high performance anion exchange chromatography with pulsed amperometric detection (HPAEC-PAD) A) 2F-Tre, B) 3F-Tre, C) 6F-Tre. 2F-Tre was detected in samples labelled with this analogue. We speculate that 3F-Tre and 6F-Tre are present, however the presence of a peak from *Mtb* control cells that runs at the same time as the 3F-Tre and 6F-Tre standards hindered our ability to confirm 3F-Tre and 6F-Tre in lipid extracts using this method. The retention times and peak areas are in Table S9.

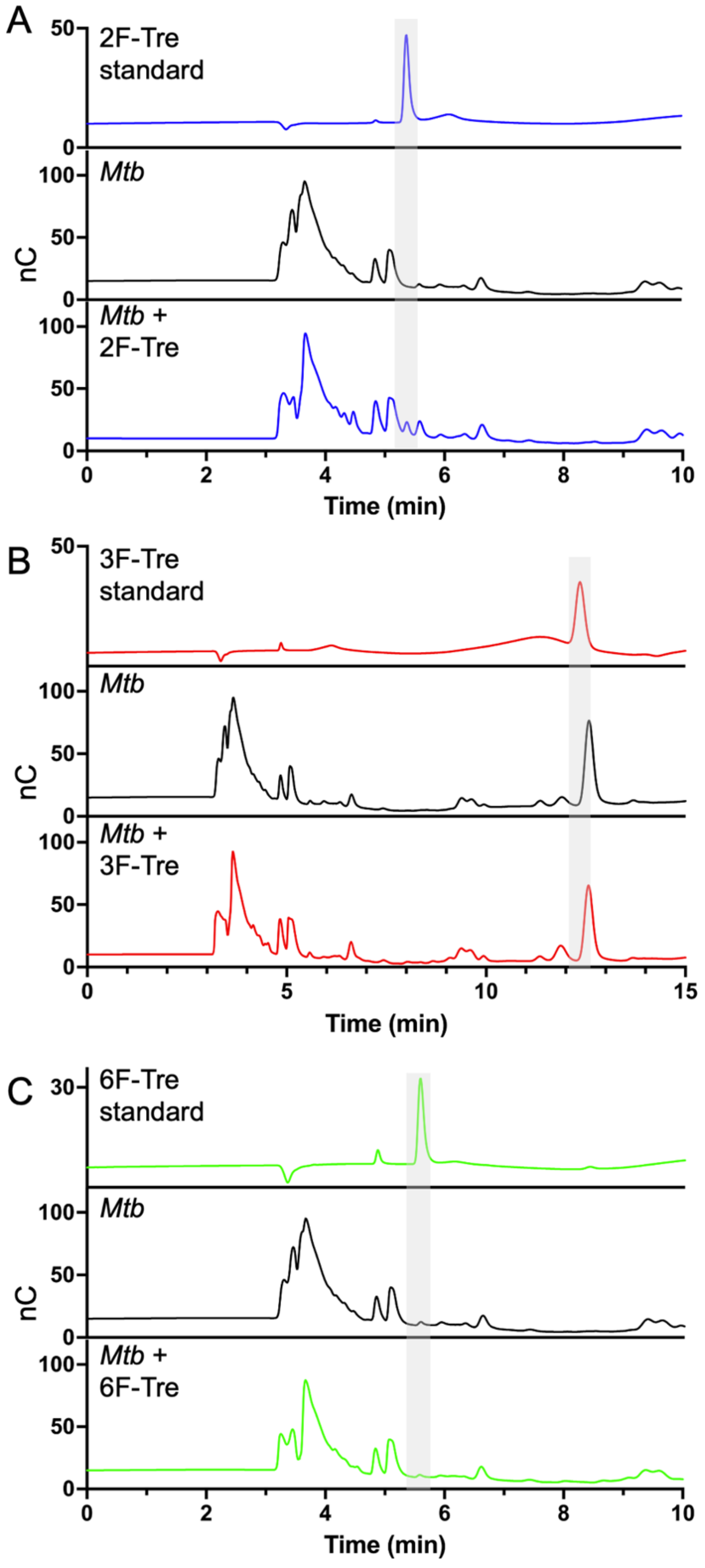

Table S9. Analysis of hydrolysed lipid extracts F-Tre labelled *Mycobacterium tuberculosis.* Retention times and peaks areas of relevant peaks in HPAEC traces (Fig. S9) are shown. Peaks corresponding to the F-Tre analogue are in bold.

	2F-Tre		3F-Tre		6F-Tre	
	Retention time (min)	Peak area (nC*min)	Retention time (min)	Peak area (nC*min)	Retention time (min)	Peak area (nC*min)
F-Tre standard	**5.367**	**3.947**	**12.359**	**6.303**	**5.575**	**2.537**
Mtb control	5.084<br>5.584	4.060<br>0.159	11.900<br>12.575	1.736<br>19.947	5.584<br>5.934	0.159<br>0.231
Mtb + F-Tre	5.075<br>**5.367**<br>5.584	3.631<br>**0.662**<br>1.209	11.875<br>12.567	3.163<br>14.605	5.584<br>5.934	0.106<br>0.067

Fig. S10. F-Glc metabolite analysis of *Mtb* labelled with F-Tre analogues. *Mtb* was cultured in the presence of (A) 2F-Tre, (B) 3F-Tre or (C) 6F-Tre (100 µM) and the cytosolic extracts analysed by high performance anion exchange chromatography with pulsed amperometric detection (HPAEC-PAD). The retention times and peak areas are in Table S10.

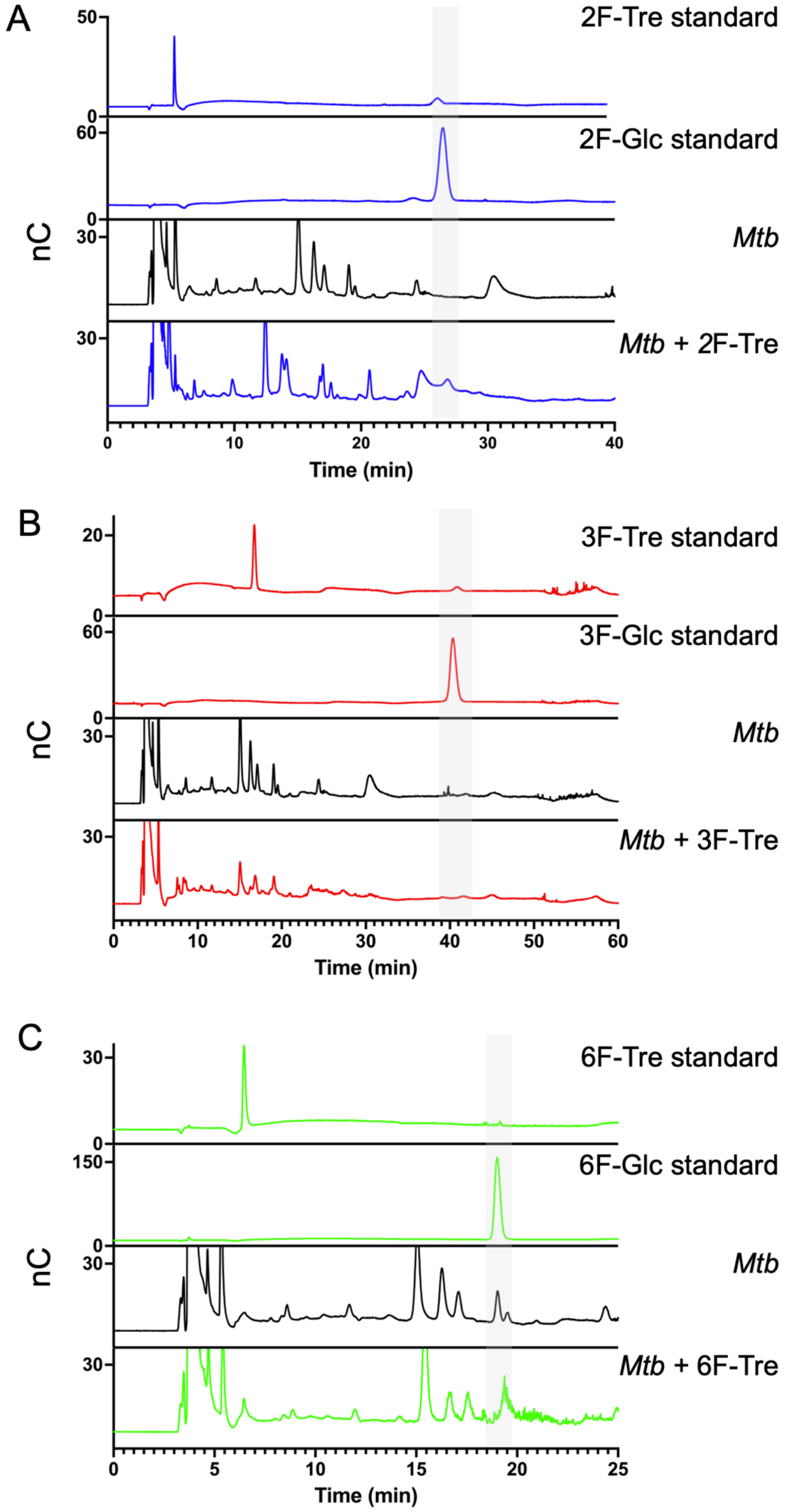

Table S10. Analysis of F-Glc metabolites. Retention times and peaks areas of relevant peaks in HPAEC traces (Fig. S10) are shown. Peaks corresponding to the F-Glc analogue are in bold.

	F-Glc standard		*Mtb* control		*Mtb* + F-Tre	
	Retention time (min)	Peak area (nC*min)	Retention time (min)	Peak area (nC*min)	Retention time (min)	Peak area (nC*min)
2F	**26.450**	**37.1714**	**-**	**-**	**26.800**	**1.9724**
3F	**40.350**	**37.3341**	39.826	0.2515	-	-
6F	**19.009**	**48.8767**	19.026<br>19.501	1.9724<br>0.5083	19.375	3.4485

Fig. S11. SIMS mass spectra of *Mtb* cells treated with 2F-Tre, 3F-Tre, 6F-Tre and non-treated control cells, from m/z 1-50. *Mtb* was grown to OD_{600} 1-1.2 in the presence of 2F-Tre, 3F-Tre or 6F-Tre (100 µM), and a non-treated control. Mass spectra recorded using a ThermoFisher Scientific Scios Ga source Dualbeam system equipped with a Hiden electrostatic quadrupole secondary ion mass spectrometer (EQS) operating in negative ion mode.

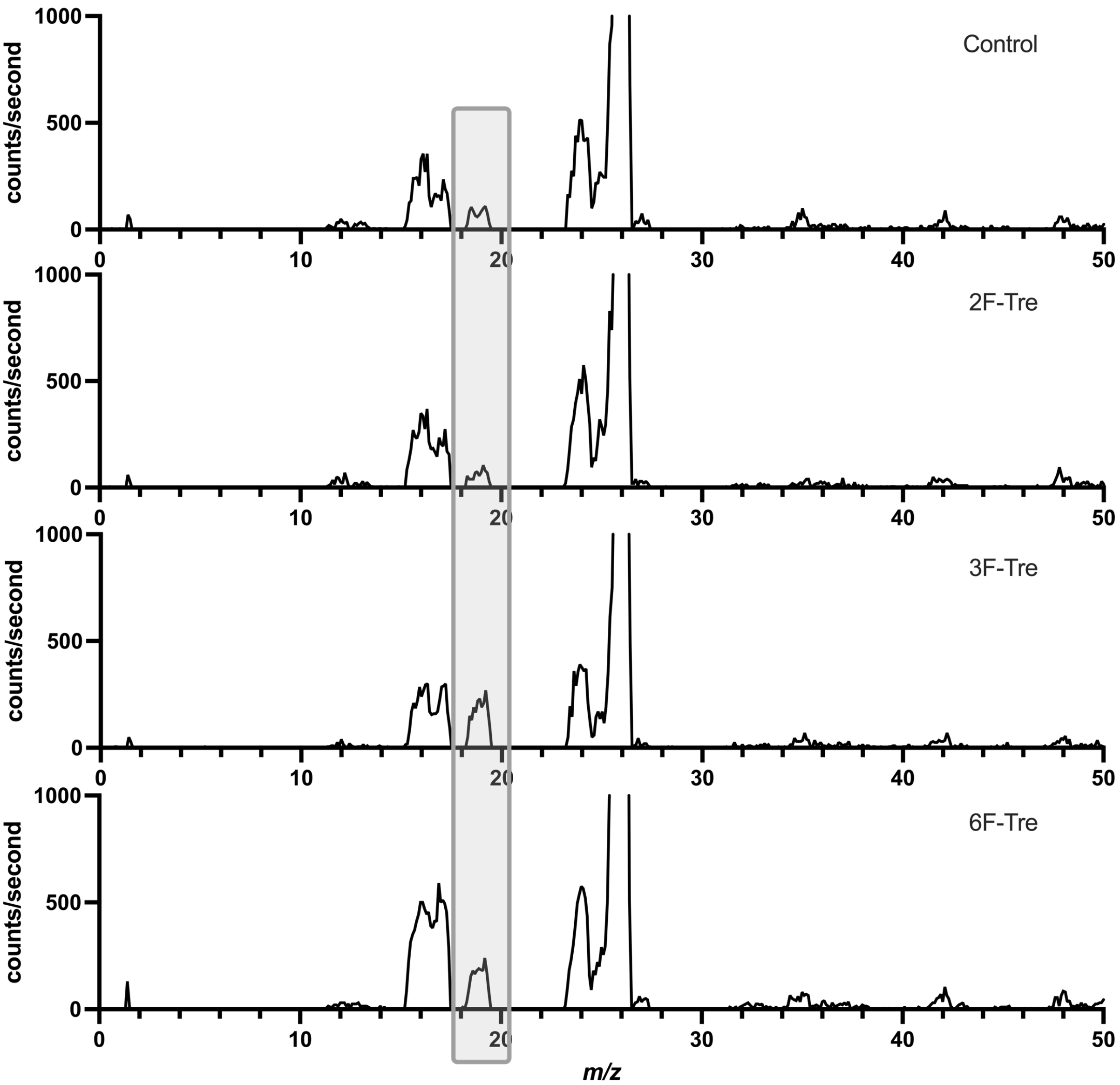

Experimental

General Information and Procedures

Unless stated, the chemicals and solvents, including anhydrous solvents, used in these syntheses were used as supplied and without further purification. 2-deoxy-2-fluoro-glucose, 3-deoxy-3-fluoro-glucose, 6-deoxy-6-fluoro-glucose and UDP-glucose were purchased from Carbosynth. Trehalose was purchased from Acros Organics. Methanol (MeOH), dichloromethane (DCM), pyridine, ethyl acetate (EtOAc), toluene, triethylamine, and magnesium sulfate ($MgSO_4$) were purchased from Fisher Scientific at laboratory reagent grade. Anhydrous *N,N*-dimethylformamide (DMF) >99.8%, anhydrous dichloromethane (DCM), deuterium oxide (D_2O) 99.9%, sodium nitrite 98% and triflic anhydride 99% were purchased from Sigma-Aldrich. Deuterochloroform ($CDCl_3$) 99.8% and deuteromethanol (MeOD) 99.8% were purchased from Apollo Scientific. Purified trehalose monomycolate (TMM), NR-48784, and purified trehalose dimycolate (TDM), NR-14844, were obtained through BEI Resources, NIAID, NIH.

^{1}H NMR, ^{13}C NMR and MS data

Proton (^{1}H), carbon (^{13}C) and fluorine (^{19}F) NMR spectra were obtained at 298 K on a Bruker DPX-400 instrument. ^{19}F NMRs were proton decoupled. NMRs were fully assigned using COSY, HSQC and HMBC. ^{1}H NMR chemical shifts are quoted in parts per million (ppm), using the residual solvent as the internal standard (^{1}H D_2O = 4.79 ppm). Coupling constants (J) are reported in hertz (Hz) with the following abbreviations: s, singlet; d, doublet; t, triplet; q, quartet; quin, quintet; m, multiplet; br, broad. Mass spectra were recorded on a Bruker Esquire 2000 spectrometer using electrospray ionisation (ESI). *M/z* values are reported in Daltons (Da).

Bacterial strains, cell lines, culture conditions and chemicals

Mycobacterium tuberculosis H37Rv and *Mycobacterium bovis* BCG (ATCC-35734) were routinely grown at 37 °C in Middlebrook 7H9 broth supplemented with 0.2% glycerol, 0.05% Tween 80 and 10% albumin-dextrose-catalase (ADC) or on Middlebrook 7H10 plates supplemented with 0.5% glycerol and 10% oleic acid-albumin-dextrose-catalase (OADC). The gene deletion mutant and complemented strains: *M. bovis BCG ΔlpqY-sugABC* and *M. bovis* BCG *ΔlpqY-sugABC*:pMV306_*lpqY-sugABC*, a gift from Professor Rainer Kalscheuer (HHU Dusseldorf, Germany), were cultured with the addition of hygromycin (50 mg/L) or hygromycin (50 mg/L) plus kanamycin (20 mg/L) respectively. PBST is phosphate buffered saline supplemented with 0.05% Tween 80. All *Mtb* work was carried out within a containment level-3 laboratory.

Expression and purification of TreT

The trehalose synthase (TreT) enzyme from *Thermoproteus tenax* was overexpressed and purified as described previously[1]. *Escherichia coli* Top 10 were transformed with the treT_pBADHisA expression plasmid (a gift from Dr B Swarts (Central Michigan University, USA)) and grown at 37 °C to an optical density at 600 nm (OD600) of 0.6-0.8 in Terrific Broth (Difco) supplemented with 100 µg/mL ampicillin. Protein production was induced with 1 mM L-arabinose and the cultures were grown at 37 °C overnight with shaking (180 rpm).

The cells were harvested (4,000 x *g*, 45 min, 4 °C) and the pellets were resuspended in PBS and frozen at -80 °C. The frozen pellets were resuspended in 50 mM NaH_2PO_4, 500 mM NaCl, 20 mM imidazole, pH 8.0 (buffer A), Complete Protease Inhibitor Cocktail (Roche) and lysozyme (60 mg) were added, and the cells sonicated on ice (MSE Soniprep 150 plus). Following sonication, the cells were centrifugated (39,000 x *g*, 30 min, 4 °C), the supernatant was filtered (0.45 µm filter, EMD Millipore) and loaded onto a pre-equilibrated HisPur Ni^{2+}-affinity resin (Thermo Scientific). The column was washed with buffer A (5 column volumes) and TreT was eluted from the Ni^{2+}resin with increasing concentrations of imidazole. Fractions containing the TreT protein were dialysed at 4 °C for 16 h (50 mM HEPES, 300 mM NaCl, pH 8.0 (buffer B) and purified further using size exclusion chromatography (Superdex 200 16/60 column (GE Healthcare)). Purified TreT fractions were pooled, and the protein concentrated to 1.5-5 mg/mL (Amicon, 10 kDa MWCO) and stored at 4 °C.

Chemoenzymatic synthesis of fluorinated trehalose derivatives

The enzymatic reaction contained either 2-deoxy-2-fluoro-D-glucose (30 mM), 3-deoxy-3-fluoro-D-glucose (30 mM) or 6-deoxy-6-fluoro-D-glucose (30 mM), UDP-Glucose (45 mM), $MgCl_2$ (40 mM), and TreT (300 µg/mL) in 50 mM HEPES, 300 mM NaCl, pH 8.0, and was incubated at 70 °C for 2 h, with shaking (300 rpm) and then cooled by placing on ice. A 10 kDa centrifugal filter unit was pre-rinsed with deionised water (2 mL) five times by centrifugation (3,200 x g for 20 min, room temperature). The cooled enzymatic reaction was then added and centrifuged at 3,200 x g for 20 min at room temperature, after which the centrifuge filter was washed 3 times with 2 mL deionised water (3,200 x g, 20 min, room temperature). The filtrates were combined and Bio-Rad Bio-Rex RG 501-X8 resin (1 g) added, and the mixture stirred at room temperature for 1 h. The mixture was then filtered, and the resin washed with 10 mL deionised water. The filtrates were combined and lyophilised to give the product as a white solid. The reactions were monitored by TLC (5:3:2 n-butanol/ethanol/water), stained with 5% H_2SO_4 in ethanol and heated to visualise spots containing sugars.

2-deoxy-2-fluoro-trehalose (2)[2]

From 19 mg of 2-deoxy-2-fluoro-glucose obtained 16.0 mg (45%) of 2-deoxy-2-fluoro-trehalose as a white solid. ^1H NMR (400 MHz, D_2O) δ 5.42 (1H, d, J = 4.0 Hz, H^1), 5.20 (1H, d, J = 3.5 Hz, $H^{1'}$), 4.50 (1H, ddd, J = 4.0, 9.5 Hz, $J_{H,F}$ = 49.0 Hz, H^2), 4.11 (1H, dt, J = 9.5 Hz, $J_{H,F}$ = 13.5 Hz, H^3), 3.69 – 3.94 (7H, m, $H^{3'}$, H^5, $H^{5'}$, H^{6ab}, $H^{6ab'}$), 3.65 (1H, dd, J = 3.5, 10.5 Hz, $H^{2'}$), 3.50 (1H, t, J = 9.5 Hz, H^4), 3.44 (1H, t, J = 9.5 Hz, $H^{4'}$). ^{13}C NMR (100 MHz, D_2O) δ 94.0 (C1'), 91.2 (d, $J_{C,F}$ = 21.5 Hz, C^1), 89.5 (d, $J_{C,F}$ = 188 Hz, C^2), 72.6 ($C^{5'}$), 72.2 (C^5), 71.1 (d, $J_{C,F}$ = 17.0 Hz, C^3), 70.9 ($C^{2'}$), 69.6 ($C^{4'}$), 69.1 ($C^{3'}$), 69.0 (C^4), 60.47, 60.27 (C^6 and $C^{6'}$). ^{19}F NMR (400 MHz, D_2O) δ -201.1. *m/z* (ES^-): $[M-H]^-$ calcd. for $C_{12}H_{21}O_{10}^-$, 343.1; found 343.1.

3-deoxy-3-fluoro-trehalose (3)[2]

From 25 mg of 3-deoxy-3-fluoro-glucose obtained 19.6 mg (42%) of 3-deoxy-3-fluoro-trehalose as a white solid. ^1H NMR (400 MHz, D_2O) δ 5.25 (1H, t, J = 4.0 Hz, H^1), 5.19 (1H, d, J = 4.0 Hz, H1'), 4.65 – 4.87 (1H, m, H^3), 3.94 (1H, ddd, J = 4.0, 9.5 Hz, $J_{H,F}$ = 13.0 Hz, H^2), 3.69 – 3.90 (8H, m, $H^{3'}$, H^4, H^5, $H^{5'}$, H^{6ab}, $H^{6ab'}$), 3.65 (1H, dd, J = 4.0, 10.5 Hz, $H^{2'}$), 3.46 (1H, t, J = 10.0 Hz, $H^{4'}$). ^{13}C NMR (100 MHz, D_2O) δ 94.4 (d, $J_{C,F}$ =

170 Hz, C^3), 93.44, 93.41 (C^1 and $C^{1'}$), 72.5 ($C^{3'}$), 72.2 ($C^{5'}$), 71.65 (d, $J_{C,F}$ = 7.0 Hz, C^5), 70.9 ($C^{2'}$), 69.6 ($C^{4'}$), 69.5 (d, J = 25.0 Hz, C^2), 67.85 (d, $J_{C,F}$ = 18.0 Hz, C^4), 60.5, 60.0 (C^6 and $C^{6'}$). ^{19}F NMR (400 MHz, D_2O) δ -199.7. *m/z* (ES^-): $[M-H]^-$ calcd. for $C_{12}H_{21}O_{10}^-$, 343.1; found 343.1.

6-deoxy-6-fluoro-trehalose (5)[2]

From 19 mg of 6-deoxy-6-fluoro-glucose obtained 17.3 mg (49%) of 6-deoxy-6-fluoro-trehalose as a white solid. ^{1}H NMR (400 MHz, D_2O) δ 5.22 (1H, d, J = 4.0 Hz, H^1), 5.19 (1H, d, J = 4.0 Hz, $H^{1'}$), 4.58 – 4.78 (2H, m, H^{6ab}), 3.98 (1H, dd, J = 10.0 Hz, $J_{H,F}$ = 29.0 Hz, H^5), 3.81 – 3.91 (4H, m, H^3, $H^{3'}$, $H^{5'}$, $H^{6a'}$), 3.77 (1H, dd, J = 5.5, 12.0 Hz, $H^{6b'}$), 3.66 (2H, td, J = 4.0, 10.5 Hz, H^2 and $H^{2'}$), 3.56 (1H, t, J = 9.5 Hz, H^4), 3.46 (1H, t, J = 9.5 Hz, $H^{4'}$), ^{13}C NMR (100 MHz, D_2O) δ 93.6, 93.5 (C^1 and $C^{1'}$), 82.07 (d, $J_{C,F}$ = 168 Hz, C^6), 72.5 (C^3), 72.3 ($C^{3'}$), 72.2 ($C^{5'}$), 71.0 (d, $J_{C,F}$ = 10.5 Hz, C^5), 70.95, 70.88 (C^2 and $C^{2'}$), 69.6 ($C^{4'}$), 68.5 (d, $J_{C,F}$ = 6.5 Hz, C^4), 60.5 ($C^{6'}$). ^{19}F NMR (400 MHz, D_2O) δ -235.5. *m/z* (ES^-): $[M-H]^-$ calcd. for $C_{12}H_{21}O_{10}^-$, 343.1; found 343.1.

Chemical synthesis of 4-deoxy-4-fluoro-trehalose (Scheme S1)

2,3,6,2′,3′,4′,6’,-hepta-*O*-benzoyl-α,α’-D-trehalose (6)[2]

Trehalose dihydrate (5 g, 13.2 mmol) was suspended in pyridine (75 mL) under nitrogen and cooled to -40 °C. Benzoyl chloride (11.5 mL, 99.1 mmol) was added dropwise, and the reaction was maintained at -40 °C for 2 h before allowing to warm slowly to room temperature and stirred for 16 h. A further portion of benzoyl chloride was added (1.54 mL, 13.2 mmol) and the reaction stirred at room temperature for a further 20 h. The reaction mixture was poured into ice cold 1 M HCl (100 mL) and extracted with EtOAc (3 x 80 mL). The combined organics extracts were washed with sat. $NaHCO_3$ (2 x 80 mL) and brine (80 mL). The organic phase was dried ($MgSO_4$), filtered and concentrated *in vacuo* to give the crude product, which was purified by column chromatography (9:1, toluene/EtOAc) to give the desired product as a white foam (1.95 g, 14 %). ^{1}H NMR (400 MHz, CDCl3) δ 7.81 – 8.13 (14H, m, ArH), 7.22 – 7.62 (21H, m, ArH), 6.28 (1H, t, J = 10.0 Hz, H^3), 5.98 (1H, t, J = 9.5 Hz, $H^{3'}$), 5.68 – 5.71 (2H, m, H^1, H^4), 5.64 (1H, d, J = 4.0 Hz, $H^{1'}$), 5.50 (1H, dd, J = 10.5, 4.0 Hz, H^2), 5.46 (1H, dd, J = 10.0, 4.0 Hz, $H^{2'}$), 4.37 (1H, ddd, J = 10.5, 4.5, 3.0 Hz, H^5), 4.22 (1H, dd, J = 12.5, 4.0 Hz, $H^{6a'}$), 4.08 (1H, ddd, J = 10.0, 4.0, 2.0 Hz, $H^{5'}$), 3.98 (1H, dd, J = 12.5, 3.0 Hz, H^{6a}), 3.85 – 3.91 (2H, m, H^{6b}, $H^{6b'}$), 3.82 (1H, t, J = 10.0 Hz, $H^{4'}$); ^{13}C NMR (100 MHz, CDCl3) δ 167.3, 167.0, 165.9, 165.7, 165.5, 165.0 (C=O), 134.1, 133.9, 133.6, 133.60, 133.5, 133.3, 133.2, 130.3, 130.03, 129.99, 129.96, 129.89, 129.85, 129.83, 129.5, 129.4, 129.2, 129.1, 128.9, 128.80, 128.76, 128.57, 128.54, 128.47, 128.43 (ArC), 93.1, 92.9(C^1, $C^{1'}$), 73.6, 71.3, 71.2, 70.8, 70.3, 69.3, 68.9, 68.7 (C^2, C^3, C^4, C^5, $C^{2'}$, $C^{3'}$, $C^{4'}$, $C^{5'}$), 62.5, 62.0 (C^6, $C^{6'}$); *m/z* (ES^+): $[M+Na]^+$ calcd. for $C_{61}H_{50}O_{18}Na^+$, 1093.3; found 1093.3.

2,3,6,-tri-*O*-benzoyl-α-D-galactopyranosyl-(1→1)-2′,3′,4′,6′,-tetra-*O*-benzoyl-α-D-glucopyranoside (8)[2]

2,3,6,2′,3′,4′,6,-Hepta-*O*-benzoyl-α,α-D-trehalose (**6**) (1.95 g, 1.82 mmol) was dissolved in DCM (30 mL) under nitrogen and cooled to 0 °C. Pyridine (1.47 mL, 18.2 mmol) and triflic anhydride (613 μL, 3.64 mmol) were added and the reaction allowed to slowly warm to room temperature and stirred for 3 h. The reaction

mixture was diluted with DCM (20 mL) and washed with 1 M HCl (50 mL), sat. $NaHCO_3$ (50 mL) and water (50 mL). The organic phase was dried ($MgSO_4$), filtered and concentrated *in vacuo* to give the intermediate triflate as a white solid, which was dissolved in DMF (16 mL) under nitrogen and sodium nitrite (629 mg, 9.11 mmol) was added. The reaction was stirred at room temperature for 16 h. A further portion of sodium nitrite (251 mg, 3.64 mmol) was added, and the reaction stirred for a further 6 h. The reaction was diluted with DCM (60 mL) and washed with water (4 x 60 mL). The organic phase was dried ($MgSO_4$), filtered and concentrated *in vacuo* to give the crude product which was purified by column chromatography (9:1, toluene/EtOAc) to give the desired product as a white solid (670 mg, 34 %). ^{1}H NMR (400 MHz, $CDCl_3$) δ 7.73 – 8.14 (14H, m, Ar<u>H</u>), 7.20 – 7.63 (21H, m, Ar<u>H</u>), 6.25 (1H, t, *J* = 10.0 Hz, $\underline{H}^3$), 5.85 – 5.97 (2H, m, $H^{2'}$, $H^{3'}$), 5.75 (1H, d, *J* = 4.0 Hz, H^1), 5.72 (1H, d, *J* = 3.0 Hz, $H^{1'}$), 5.65 (1H, t, *J* = 10.0 Hz, H^4), 5.47 (1H, dd, *J* = 10.0, 4.0 Hz, H^2), 4.18 – 4.38 (4H, m, H^4, H^5, $H^{5'}$, $H^{6a'}$), 3.99 – 4.08 (2H, m, H^{6a}, $H^{6b'}$), 3.94 (1H, dd, *J* = 12.5, 5.0 Hz, H^{6b}); ^{13}C NMR (100 MHz, $CDCl_3$) δ 166.2, 166.0, 165.8, 165.73, 165.65, 165.5, 165.1 (<u>C</u>=O), 133.8, 133.70, 133.65, 133.6, 133.4, 133.3, 133.2, 130.0, 129.99, 129.95, 129.92, 129.87, 129.84, 129.5, 129.4, 129.3, 129.2, 129.0, 128.84, 128.81, 128.77, 128.69, 128.58, 128.47, 128.46, 128.42 (Ar<u>C</u>), 93.1, 92.4 (C^1, $C^{1'}$), 71.4, 70.8, 70.4, 69.0, 68.61, 68.58, 68.2, 67.5 (C^2, C^3, C^4, C^5, $C^{2'}$, $C^{3'}$, $C^{4'}$, $C^{5'}$), 62.35, 62.18 (C^6, $C^{6'}$); *m/z* (ES^+): $[M+Na]^+$ calcd. for $C_{61}H_{50}O_{18}Na^+$, 1093.3; found 1093.3.

4-fluoro-2,3,6,-tri-*O*-benzoyl-α-D-glucopyranosyl-(1→1)-2′,3′,4′,6′,-tetra-*O*-benzoyl-α-D-glucopyranoside (9)[2]

2,3,6,-tri-*O*-benzoyl-α-D-galactopyranosyl-(1→1)-2′,3′,4′,6′,-tetra-*O*-benzoyl-α-D-glucopyranoside (**8**) (331 mg, 0.309 mmol) was dissolved in anhydrous dichloromethane (10 mL) under nitrogen at room temperature. Diethylaminosulfur trifluoride (DAST, 102 μL, 0.773 mmol) was then added dropwise. The solution was heated to 40 °C for 72 h. The product was diluted with dichloromethane (20 mL), then washed with $NaHCO_3$ (2 x 30 mL) and water (30 mL). The organic layer was dried over anhydrous $MgSO_4$, filtered, and concentrated *in vacuo*. The product was purified by column chromatography on a Biotage Selekt with an Sfär cartridge (10 g, silica – 60 μm) (1-20% ethyl acetate in toluene) to give the fluorinated intermediate (208 g, 63%). ^{1}H NMR (400 MHz, CDCl3) δ 7.79 – 8.11 (14H, m, ArH), 7.18 – 7.63 (21H, m, ArH), 6.19 – 6.38 (2H, m, H^3, $H^{3'}$), 5.63 – 5.73 (3H, m, H^1, $H^{1'}$, $H^{4'}$), 5.51 (1H, dd, *J* = 10.5, 4.0 Hz, H^2 or $H^{2'}$), 5.41 (1H, dd, *J* = 10.0, 4.0 Hz, H^2 or $H^{2'}$), 4.74 (1H, dt, *J* = 9.4 Hz, $J_{H,F}$ = 50.5 Hz, H^4), 4.21 – 4.39 (2H, m, H^5, $H^{5'}$), 3.91 – 4.05 (3H, m, H^{6a}, $H^{6a'}$,$H^{6b'}$), 3.84 (1H, dd, *J* = 12.5, 4.5 Hz, H^{6b}). ^{13}C NMR (100 MHz, CDCl3) δ 165.9, 165.49, 165.47, 165.41, 165.36, 165.0 (<u>C</u>=O) 134.1, 133.9, 133.6, 133.5, 133.3, 133.24, 133.15, 129.94, 129.90, 129.79, 129.76, 129.4, 129.2, 129.04, 128.98, 128.94, 128.88, 128.7, 128.61, 128.59, 128.51, 128.48, 128.41, 128.37, 128.28, 128.21 (Ar<u>C</u>), 93.0, 92.7 (C^1, $C^{1'}$), 86.9 (d, $J_{C,F}$ = 189.0 Hz, C^4), 71.1, 70.7 (d, $J_{C,F}$ = 8.0 Hz), 70.4 (d, $J_{C,F}$ = 20.5 Hz), 70.1, 68.72, 68.69, 67.9 (d, $J_{C,F}$ = 23.0 Hz). (C^2, C^3, C^5, $C^{2'}$, $C^{3'}$, $C^{4'}$, $C^{5'}$), 61.9, 61.6 (C^6, $C^{6'}$). ^{19}F NMR (400 MHz, D_2O) δ -197.4 *m/z* (ES^+): $[M+Na]^+$ calcd. for $C_{61}H_{59}FO_{17}Na^+$, 1095.3; found 1095.3.

4-deoxy-4-fluoro-trehalose (4)[2]

4-fluoro-2,3,6,-tri-*O*-benzoyl-α-D-glucopyranosyl-(1→1)-2′,3′,4′,6′,-tetra-*O*-benzoyl-α-D-glucopyranoside (**9**) (208 mg, 0.194 mmol) was dissolved in 0.2 M methanolic NaOMe (5 mL) and the reaction was stirred at room temperature for 14 h. Amberlite® IR120 acidic resin (200 mg) was then added and the mixture stirred at room temperature for 30 min to neutralise the reaction, filtered and concentrated *in vacuo*. The residue was taken up in water (10 mL), washed with petroleum ether (40-60 °C) (3 x 10 mL) and then lyophilised to give the product as a white solid (63.1 mg, 95%); ^{1}H NMR (400 MHz, D_2O) δ 5.20 (2H, d, J = 4.0 Hz, H^1 and $H^{1'}$), 4.38 (1H, dt, J = 9.5 Hz, $J_{H,F}$ = 51.0 Hz, H^4), 4.16 (1H, dt, J = 9.5 Hz, $J_{H,F}$ = 16.0Hz, H^3), 3.98 – 4.07 (1H, m, H^5), 3.73 – 3.92 (6H, m, $H^{3'}$, $H^{5'}$, H^{6ab}, $H^{6ab'}$), 3.70 (1H, dd, J = 4.0, 10.0 Hz, H^2), 3.65 (1H, dd, J = 4.0, 10.0 Hz, $H^{2'}$), 3.46 (1H, t, J = 9.5 Hz, $H^{4'}$). ^{13}C NMR (100 MHz, D_2O) δ 93.5 ($C^{1'}$), 93.2 (C^1), 89.1 (d, J = 179.5 Hz, C^4), 72.5, 72.2 (C3’ and C5’), 71.0 ($C^{2'}$), 70.7 (d, J = 17.5 Hz, C^3), 70.5 (d, J = 8.5 Hz, C^2), 69.7 ($C^{4'}$), 69.60 (d, J = 23.5 Hz, C^5), 60.5, 59.8 (C^6 and $C^{6'}$). ^{19}F NMR (400 MHz, D_2O) δ -198.3. *m/z* (ES^-): $[M-H]^-$ calcd. for $C_{12}H_{21}O_{10}^-$, 343.1; found 343.1.

Production and purification of *Mtr* LpqY

Mtr LpqY was overexpressed and produced as described previously[3]. In brief, *E. coli* BL21 (DE3) cells containing the *mtr_lpqY_sumo* plasmid were grown at 27 °C to an OD_{600} of 0.4 to 0.6 in Terrific broth medium supplemented with 50 µg/mL kanamycin. Protein production was induced with 1 mM isopropyl-β-thiogalactopyranoside, and the cultures were grown at 16 °C overnight with shaking (180 rpm). The cells were harvested (4,000 x *g*, 45 min, 4 °C) and resuspended in lysis buffer (20 mM Tris, 300 mM NaCl, 10% glycerol, pH 7.5 (buffer 1)) and frozen at −80 °C. A complete protease inhibitor tablet (Roche), $MgCl_2$ (5 mM), DNase (2 mg), and lysozyme (20 mg) were added to the resuspended pellet and sonicated on ice (MSE Soniprep 150 plus). Following centrifugation (39,000 x *g*, 45 min, 4 °C), the supernatant was filtered (0.45 µm filter) and loaded onto a pre-equilibrated HisPur Ni^{2+}-NTA affinity resin (Thermo Scientific). The column was washed with buffer 1 and *Mtr* LpqY was eluted with increasing concentrations of imidazole. Fractions containing *Mtr* LpqY were digested with His-tagged SUMO protease (1 h, 30 °C, 300 µg) and dialyzed at 4 °C for 12 h against buffer 1. A second HisPur Ni^{2+}-NTA affinity resin purification step was undertaken, and fractions containing *Mtr* LpqY were pooled and purified further using size exclusion chromatography (Superdex 200 16/600 column, GE Healthcare) with 50 mM HEPES, 300 mM NaCl pH 7.5. Purified *Mtr* LpqY was concentrated to 5 to 14 mg/mL (Vivaspin 20; GE Healthcare) and stored at −80 °C.

Microscale Thermophoresis

Mtr LpqY (2.6 µM) was labelled with the amine reactive RED-NHS dye (3 µM) (second generation, NanoTemper Technologies). Following incubation in the dark for 30 min with shaking (300 rpm, room temperature), excess dye was removed using a Zeba desalting spin column (7KMWCO). The trehalose analogues were prepared in PBS containing 0.05% Tween 20, and the final concentration of the protein in the assay was 500 nM. The samples were loaded into the MonoLith NT.115 standard treated capillaries and incubated for 10 min before analysis using the Monolith NT.115 instrument (NanoTemper Technologies) at

21 °C using the auto-select excitation power (20%) and medium laser power. The binding affinities were calculated using a single-site binding model using the MST Analysis software (version 7.0). All experiments were carried out in triplicate.

STD-NMR

All the STD NMR experiments were carried out in PBS D_2O buffer, pH 7.4. The protein concentration was 28 µM and the ligand concentration (2F-Tre, 3F-Tre, 4F-Tre or 6F-Tre) was 1 mM. STD NMR spectra were acquired on a Bruker Avance III 700.25 MHz at 278 K for studies with 2F-Tre and 4F-Tre, whereas the temperature was 303 K for studies with 3F-Tre and 6F-Tre. The on- and off-resonance spectra were acquired using a train of 50 ms Gaussian selective saturation pulses using a variable saturation time from 0.5 s to 6 s, and a relaxation delay (D1) of 6 s. The residual protein resonances were filtered using a T1ρ-filter of 25 ms. All the spectra were acquired with a spectral width of 9 kHz and 24K data points using 256 scans in saturation times of 0.5, 0.75, 1, 1.25 s, 128 scans in 1.5, 2 s and 64 scans in 2.5, 3, 4, 5, 6 s. The on-resonance spectra were acquired by saturating aliphatic hydrogens, specifically at 0.53 ppm for studies with 2F-Tre and 4F-Tre, whereas irradiation was at 0.84 ppm for studies with 3F-Tre and 6F-Tre, but also by saturating of aromatics hydrogens, specifically at 7.0 ppm for studies with 2F-Tre and 4F-Tre, whereas it was 7.24 ppm for studies with 3F-Tre and 6F-Tre, where average chemical shifts used came from those predicted from shiftX2[4] for the aliphatic and aromatic residues present in the binding site of Mtr LpqY, whereas the off-resonance spectra were in all cases acquired by saturating at 40 ppm. To get accurate structural information from the STD NMR data and to minimize any T_1 relaxation bias, the STD build-up curves were fitted to the equation $STD(t_{sat}) = STDmax*(1-exp(-k_{sat}*t_{sat}))$, calculating the initial growth rate STD_0 factor as STDmax*ksat and then normalizing all of them to the highest value. DEEP-STD factors were obtained as previously described [5] with all saturation times (0.5, 0.75, 1, 1.25, 1.5, 2, 2.5, 3, 4, 5 and 6 s) for 2F-Tre, 4F-Tre and 6F-Tre and at a single saturation time (6 s) for 3F-Tre on aliphatic or aromatic regions (0.53 or 7 ppm for 2F-Tre and 4F-Tre, whereas 0.84 or 7.24 ppm 3F-Tre and 6F-Tre).

Molecular dynamics

Input preparation and equilibration

The initial coordinates of the four *Mtr* LpqY–fluorinated trehalose complexes were built from the coordinates of the model of *Mtr* LpqY bound to 6-deoxy-6-azido-trehalose[3] by manually modifying the substituents of the trehalose ligand with *Pymol*. The MD simulation setup and equilibration were performed with the BioExcel Building Blocks (*BioBB*) library[6]. The ligands were parametrized and minimized using the *acpype* and *babel* modules, respectively, of BioBB (*biobb_chemistry.acpype* and *biobb_chemistry.babel*). The minimization of the ligands was performed with the steepest descent method and the GAFF force field. The topology of the complexes were generated with the *biobb_amber.leap* module, using the ff14SB force field[7] for the protein and GAFF[8] for the ligand. Subsequently, they were minimized with the *biobb_amber.sander* module using first positional restraints of 50 kcal/mol·Å^2 on the protein heavy atoms and, secondly, positional restraints of 500 kcal/mol·Å^2 on the ligand to avoid potential changes in ligand orientation due to protein repulsion. Then,

each protein-ligand complex was immersed in a TIP3P[9] truncated octahedron water box with a distance from the protein to the box edge of 9.0 Å and Periodic Boundary Conditions, followed by the addition of a 150 mM concentration of NaCl. This gave rise to MD simulation systems of ~ 37,000 atoms. Each solvated system was minimized using the steepest descent protocol and applying positional restraints of 15 kcal/mol·Å^2 to the ligand, followed by heating up to 300 K over 2500 steps applying the Langevin thermostat[10] with a collision frequency of 1 ps^{-1} and positional restraints on the ligand of 10 kcal/mol·Å^2 (the *biobb_amber.sander* module was used). Next, each system was subjected to NVT followed by NPT equilibration of 100 ps each. A non-bonded interactions cutoff of 10.0 Å, the SHAKE algorithm for constraining the length of bonds involving hydrogen atoms, the Langevin thermostat[10] with a collision frequency of 5 ps^{-1}, and smooth positional restraints on the ligand (5 and 2.5 kcal/mol·Å^2 for NVT and NPT, respectively) were employed. During the NPT equilibration, a pressure of 1 bar was kept constant using isotropic position scaling with a pressure relaxation time of 2 ps.

MD simulations

A 100 ns of MD production run was carried out for each complex on a AMD-Ryzen 4xGPU 3070 Computing Cluster using the *pmemd.cuda* module of AMBER 20[11]. The production dynamics was performed at a constant temperature of 300 K, by applying the Langevin thermostat[10] with a collision frequency of 1 ps^{-1}, and a constant pressure of 1 bar (using isotropic position scaling with a pressure relaxation time of 1 ps). A non-bonded interactions cutoff of 9.0 Å, periodic boundary conditions (PBC)[12], and the Particle Mesh Ewald method[13] (PME) to account for the long range electrostatic effect were employed. The SHAKE algorithm[14, 15] was also employed, thus allowing 2 fs between time steps. Trajectory coordinates were saved every nanosecond. The analysis of the MD trajectories was performed using the *cpptraj* module (version *4.25.6*) of AMBER 20.[11] The evolution of protein and ligand RMSD over the simulation time was calculated against the first frame of the trajectory. For the protein RMSD, only the backbone atoms were considered for the fit and the calculation. For the ligand, we first aligned the trajectory against the first frame using the protein backbone atoms within 5 Å of the ligand as fitting selection, and subsequently, the RMSD of the ligand backbone was calculated in-place (no superposition) allowing the orientational changes and dynamics of the ligand in the binding site to be determined.

Analysis of F-Tre uptake at a single-time point

M. tuberculosis or M. bovis BCG were cultured in the presence of 2F-Tre, 3F-Tre, 4F-Tre or 6F-Tre (25 μM, 50 μM, 100 μM or 200 μM final concentration) in 5 mL culture volumes, with a starting OD_{600} of 0.05. Controls with either the equivalent volume of water or the equivalent concentration of trehalose added were also prepared. Cultures were grown until the optical density at 600 nm (OD_{600}) was between 1.0 and 1.2 and the cells then harvested by centrifugation (2,916 *x g*, 22 °C for 5 min) and washed three times (3 x 5 mL PBST). The pellets were then resuspended in 1 mL H_2O and lysed by mechanical disruption using 0.1 mm zirconia/silica beads (BioSpec Products) on a FastPrep (MP Biomedicals) ribolyser (4 x 45 s cycles with 90 s on ice in between). Samples were then centrifuged (16,200 x *g*, 10 min, 22 °C) and the supernatant collected,

lyophilised, resuspended in 1 mL 18 MΩ H_2O and filtered through a 10-kDa molecular weight cut-off centrifuge filter (Amicon) and the filtrate analysed. HPAEC-PAD was performed on a Dionex ICS5000+ system with a CarboPac PA-20 analytical column (3 mm x 150 mm) and PA-20 guard column (3 mm x 30 mm) kept at 20 °C. Pulsed amperometry with standard quadrupole waveform was used for detection. The system was equipped with an autosampler that was set up to inject 10 µL sample volumes. Multistep gradient elution was performed using a KOH eluent generation cartridge with the gradient conditions shown in Table S11. Authentic standards of trehalose, 2F-Tre, 3F-Tre, 4F-Tre and 6F-Tre were prepared at 100 µM. Chromeleon 7 software (Dionex) was used for data processing.

Table S11: High performance anion exchange chromatography KOH elution gradient for F-Tre analysis

Time (min)	KOH conc. (mM)	Flow rate (mL/min)
0	5	0.15
3	5	0.15
4	5	0.40
18	30	0.40
20	30	0.40
22	5	0.15

To quantify uptake, the peak area of 2F-Tre, 3F-Tre and 6F-Tre standards at varying concentrations were measured (Chromeleon 7 software). The peak area was plotted against concentration and simple linear regression was plotted. To determine the concentration of F-Tre analogues in cytosolic samples the area of the peaks of interest was measured (Chromeleon 7 software) and the concentration determined from the calibration plot.

Time dependent F-Tre uptake

M. tuberculosis was grown to an OD_{600} of 0.8, then 2F-Tre, 3F-Tre, 4F-Tre or 6F-Tre were added to a final concentration of 100 µM. Controls with the equivalent volume of water or equivalent concentration of trehalose were also prepared. 5 mL aliquots were taken at T=0, 0.5, 1, 1.5, 2, 3, 4, 6, 8 h and the cells were harvested by centrifugation (2,916 *x g*, 5 min, 22 °C) and washed (5 mL PBST) three times. The pellets were then resuspended in 1 mL H_2O and lysed by mechanical disruption using 0.1 mm zirconia/silica beads (BioSpec Products) on a FastPrep (MP Biomedicals) ribolyser (4 x 45 s cycles with 90 s on ice in between). Samples were then centrifuged (16,200 x *g*, 10 min, 22 °C) and the supernatant collected, lyophilised, resuspended in 1 mL 18 MΩ H_2O and filtered through a 10-kDa molecular weight cut-off centrifuge filter (Amicon). The filtrate was analysed by HPAEC-PAD and quantified as described above.

Analysis of F-Glc metabolites

M. tuberculosis was cultured in the presence of 2F-Tre, 3F-Tre, or 6F-Tre (100 μM final concentration) in 5 mL culture volumes, with a starting OD_{600} of 0.05. Controls with the equivalent volume of water were also prepared. Cultures were grown until the OD_{600} was between 1.0 and 1.2 and the cells then harvested by centrifugation (2,916 *x g*, 22 °C for 5 min) and washed three times (3 x 5 mL PBST). The pellets were resuspended in 1 mL H_2O and lysed by mechanical disruption using 0.1 mm zirconia/silica beads (BioSpec Products) on a FastPrep (MP Biomedicals) ribolyser (4 x 45 s cycles with 90 s on ice in between). Samples were then centrifuged (16,200 x *g*, 10 min, 22 °C) and the supernatant collected, lyophilised, resuspended in 1 mL 18 MΩ H_2O and filtered through a 10-kDa molecular weight cut-off centrifuge filter (Amicon) and the filtrate analysed. HPAEC-PAD was performed on a Dionex ICS5000+ system with a CarboPac PA-20 analytical column (3 mm x 150 mm) and PA-20 guard column (3 mm x 30 mm) kept at 20 °C. Pulsed amperometry with standard quadrupole waveform was used for detection. The system was equipped with an autosampler that was set up to inject 10 μL sample volumes. Multistep gradient elution was performed using a KOH eluent generation cartridge with the gradient conditions shown in Table S12. Authentic standards of 2F-Glc, 3F-Glc, and 6F-Glc were prepared at 100 μM. Chromeleon 7 software (Dionex) was used for data processing.

Table S12: High performance anion exchange chromatography KOH elution gradient for F-Glc analysis

Time (min)	KOH conc. (mM)	Flow rate (mL/min)
0	5	0.15
3	5	0.15
4	5	0.40
18	30	0.40
50	30	0.40
52	5	0.15
60	5	0.15

Lipid extraction and analysis

M. tuberculosis and *M. bovis* BCG were cultured in the presence of 2F-Tre, 3F-Tre, 4F-Tre or 6F-Tre (100 μM final concentration) in 25 mL volumes. Controls with equivalent volume of water added, and the equivalent concentration of trehalose were also prepared. Cultures were grown until the OD_{600} was between 1.0 and 1.2 then cells were harvested by centrifugation (2,916 *x g*, 5 min, 22 °C) and washed (5 mL PBST) three times. The pellets were resuspended in 2 mL in MeOH–0.3% aqueous NaCl (10:1) and 2 mL petroleum ether (60-80 °C) and the samples shaken at 800 rpm at room temperature overnight. The samples were then centrifuged (2,187 x *g*, 5 min, 22 °C) and the top layer collected. A further 2 mL petroleum ether (60-80 °C) was added to the remaining bottom layer and the samples shaken at 800 rpm for 1 h. The samples were then centrifuged (2,187 x *g*, 5 min, 22 °C) and the top layer collected and combined with the previous fraction and

dried to yield the apolar lipids. 2.3 mL of chloroform/methanol/0.3% NaCl (9:10:3) was then added to the remaining lower layer and the samples shaken at 800 rpm at room temperature overnight. The samples were then centrifuged (2,187 x *g*, 5 min, 22 °C) and the supernatant collected. The pellet was then resuspended in 750 µL of chloroform/methanol/0.3% NaCl (5:10:4) and the samples shaken at 800 rpm for a further hour. The samples were then centrifuged (2,187 x *g*, 5 min, 22 °C) and the supernatant collected. The pellet was then resuspended in 750 µL of chloroform/methanol/0.3% NaCl (5:10:4) and the samples shaken at 800 rpm for a further hour. The samples were then centrifuged (2,187 x *g*, 5 min, 22 °C) and the supernatant collected. 1.3 mL chloroform and 1.3 mL 0.3% NaCl was added to the combined supernatants and then shaken at 800 rpm at room temperature for 5 min before centrifuging (2,187 x *g*, 5 min, 22 °C). The lower phase was collected and dried to yield the polar lipids. Lipids were analysed by TLC (8:2:0.2 chloroform/methanol/NH_4OH) and compared to TMM and TDM standards. Plates were visualised with 5% H_2SO_4 in ethanol followed by heating, to detect carbohydrate compounds.

Following TLC analysis the carbohydrate head groups were cleaved and analysed by HPAEC-PAD. The isolated lipid samples were resuspended in anhydrous dichloromethane (0.2 mL), treated with NaOMe/MeOH (2.0 M, 0.2 mL), and stirred vigorously for 16 h at 60 °C. The reaction was neutralized with Amberlite H^+ resin to pH 7.0, filtered, and evaporated to dryness. The residue was then resuspended in chloroform (1.0 mL) and extracted three times with 18 MΩ H_2O (1 mL). The combined aqueous layers were lyophilised and resuspended in 300 µL 18 MΩ H_2O and analysed by HPAEC- PAD as above.

Preparation of samples for focussed ion beam (FIB) secondary ion mass spectrometry (SIMs)

M. tuberculosis was cultured in the presence of 2F-Tre, 3F-Trel, 4F-Tre or 6F-Tre (100 µM) in 5 mL volumes. Controls with equivalent volume of water added, and the equivalent concentration of trehalose were also prepared. F-Tre was added at an OD_{600} of 0.05 and the cultures were grown until the OD_{600} was between 1.0 and 1.2. The cells were harvested by centrifugation (2,916 *x g*, 5 min, 22 °C) and washed (5 mL PBST) three times, resuspended in PBST (5 mL) and 0.5 mL of the sample was centrifuged (15,871 x g, 5 min, 22 °C) and the supernatant discarded. The pellet was resuspended in glutaraldehyde (2.5% in PBS, 1 mL) and incubated at 4 °C for 90 min. The samples were then centrifuged (15,871 x g, 5 min, 22 °C) and the supernatant removed. The pellet was washed 3 times with PBST (15,871 x g, 5 min, 22 °C) and twice with deionised water (15,871 x g, 5 min, 22 °C) and stored at 4 °C prior to imaging.

Scanning electron microscopy (SEM) and FIB-SIMS

Glutaraldehyde treated pellets were resuspended in 1 mL H_2O and 5 µL was then spotted onto a copper TEM grid. The sample was left to settle for 2 min, the excess liquid blotted off with filter paper and the grid was then plunge frozen in liquid ethane (cooled with liquid N_2) and lyophilised. SEM imaging was performed using a ThermoFisher Scientific Scios Dualbeam Secondary electron (SE). SEM images were acquired using accelerating voltage 2 kV and beam current 100 pA. FIB-SIMS was performed using a ThermoFisher Scientific

Scios Ga source Dualbeam system equipped with a Hiden electrostatic quadrupole secondary ion mass spectrometer (EQS). To minimise charging effects, samples were first coated with Au/Pd using a Cressington 206HR sputter coater. SIMS mass spectra were acquired by scanning an area of approx. 5 µm x 5 µm, with a dwell time of 100 ns and beam conditions 30 kV, 1 nA. Mass spectra were acquired with the EQS operating in negative ion mode, using a mass dwell time of 200 ms and step size 0.1 amu. FIB-SIMS mapping was performed using the FIB Ga^+ beam operating at 30 kV with beam current 100 pA. Operating in negative ion mode, the EQS was set to map counts of m/z = 19 ($19F^-$). FIB-SIMS mapping was performed with a pixel dwell time 1 ms with image size 800 pixels for areas approximately 50 µm x 50 µm. Image intensity of the FIB-SIMS maps correspond to counts s-1 of m/z 19 ($19F^-$), providing qualitative distribution of fluorine. Pairs of ($19F^-$) FIB-SIMS maps for positive and control samples were acquired on the same day using the same ion beam conditions. For each positive/control pair of FIB-SIMS maps, the control image colourmap was scaled so that image intensity/colours correspond to the same image intensity scale as the positive sample. FIB-SIMS map images were smoothed using a gaussian blur 3x3 smoothing filter.

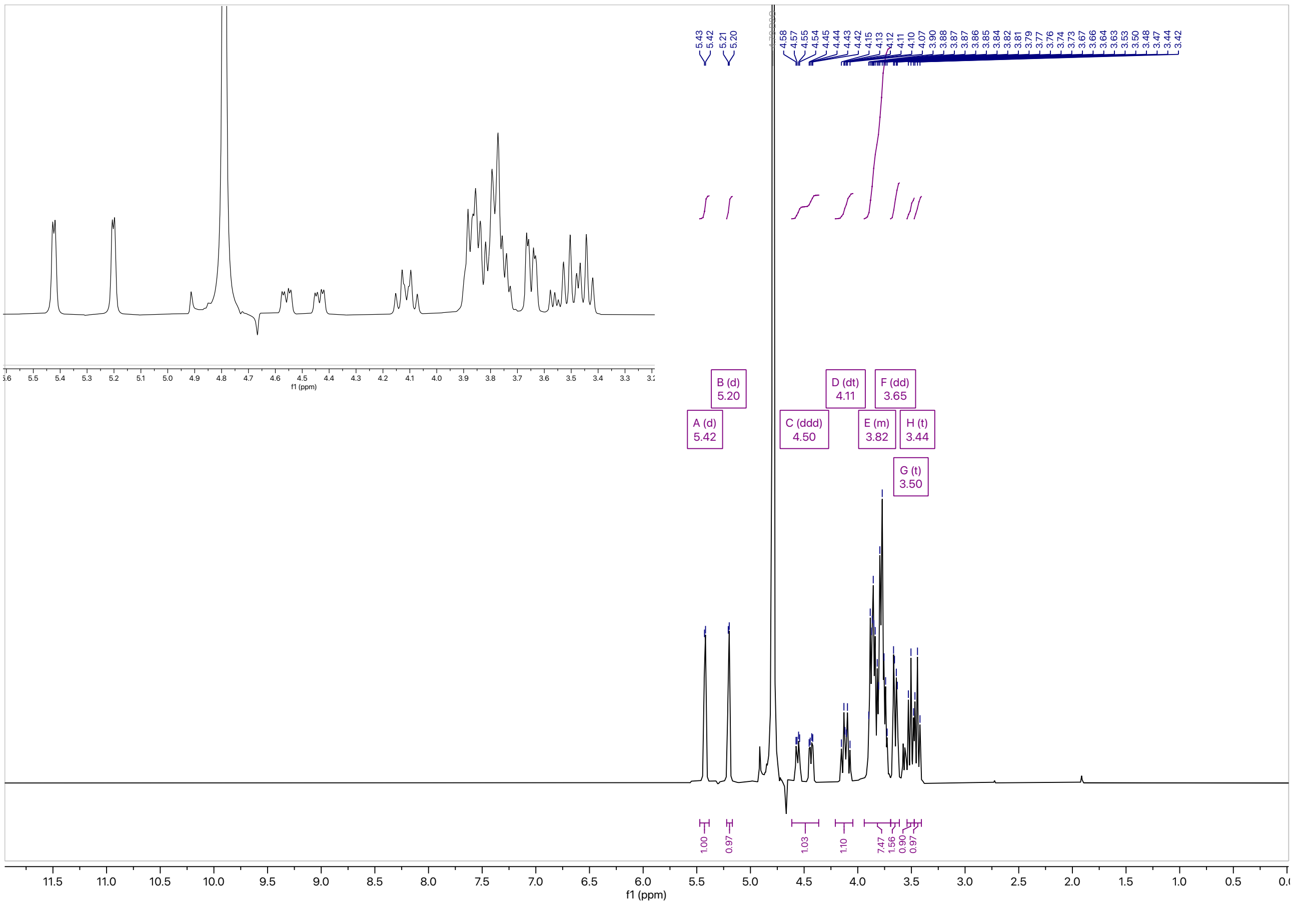

Fig. S12. ^{1}H NMR 2-deoxy-2-fluoro-trehalose (2) Inset shows the peaks zoomed in.

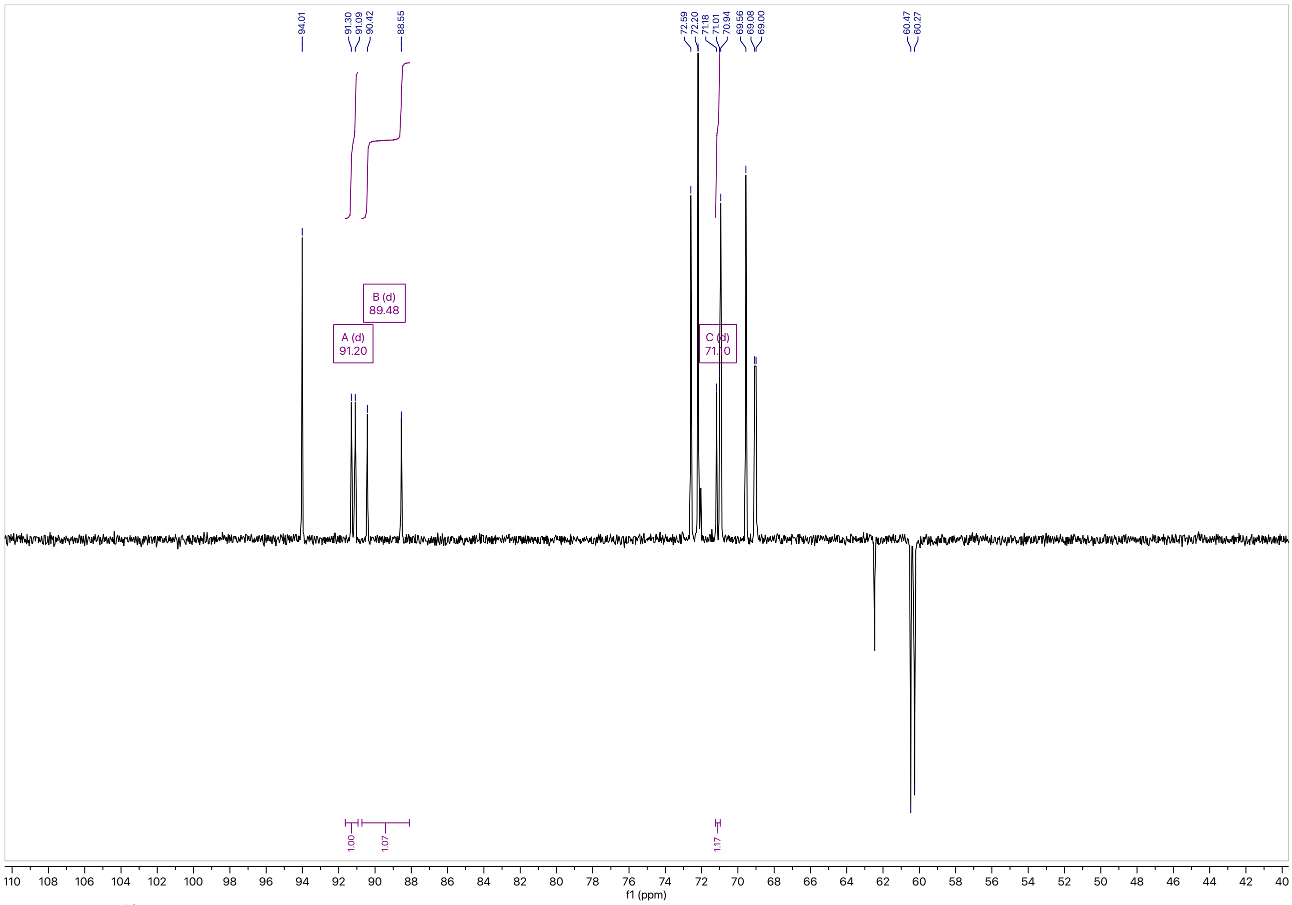

Fig. S13. ^{13}C NMR 2-deoxy-2-fluoro-trehalose (2)

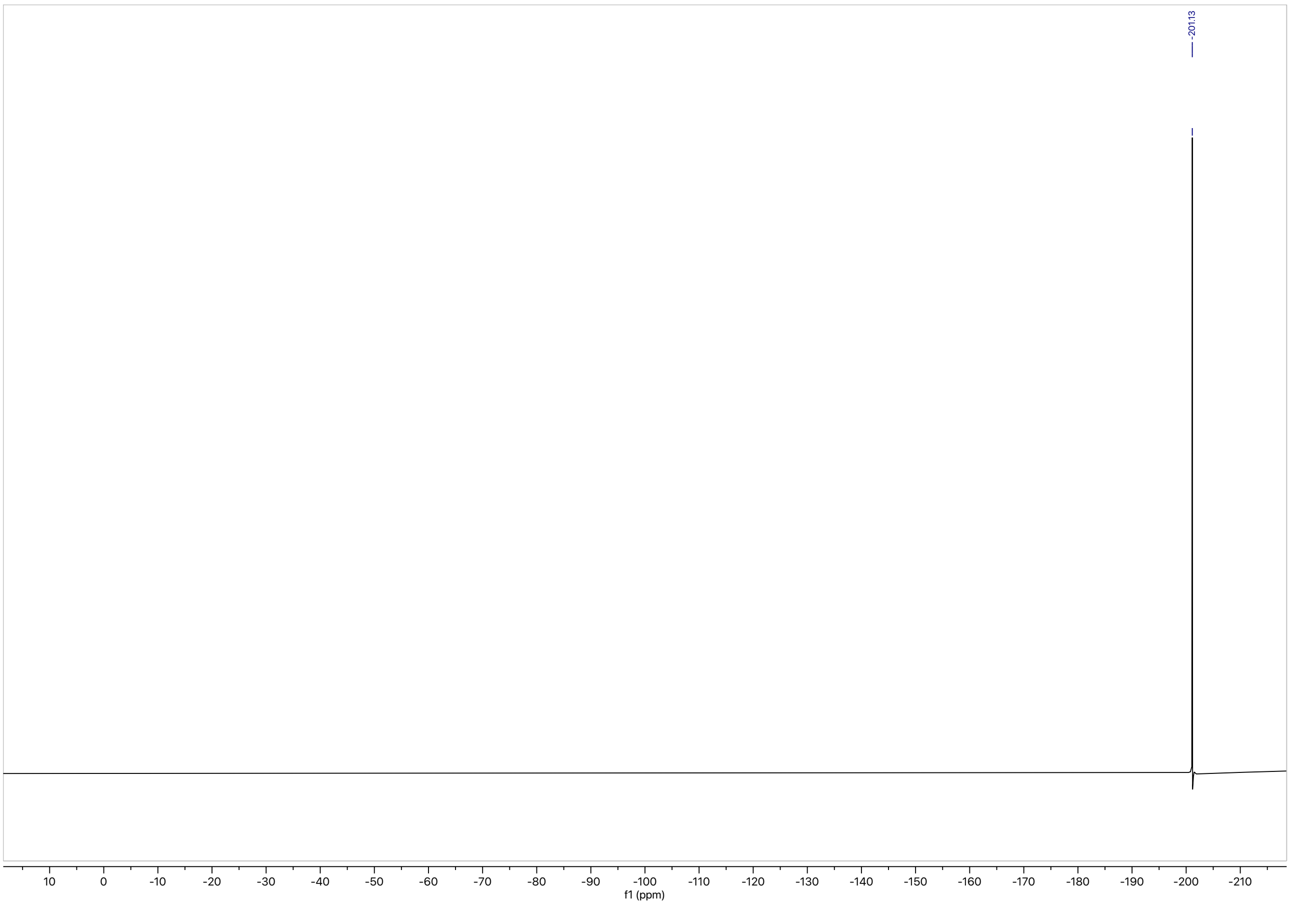

Fig. S14. ^{19}F NMR 2-deoxy-2-fluoro-trehalose (2)

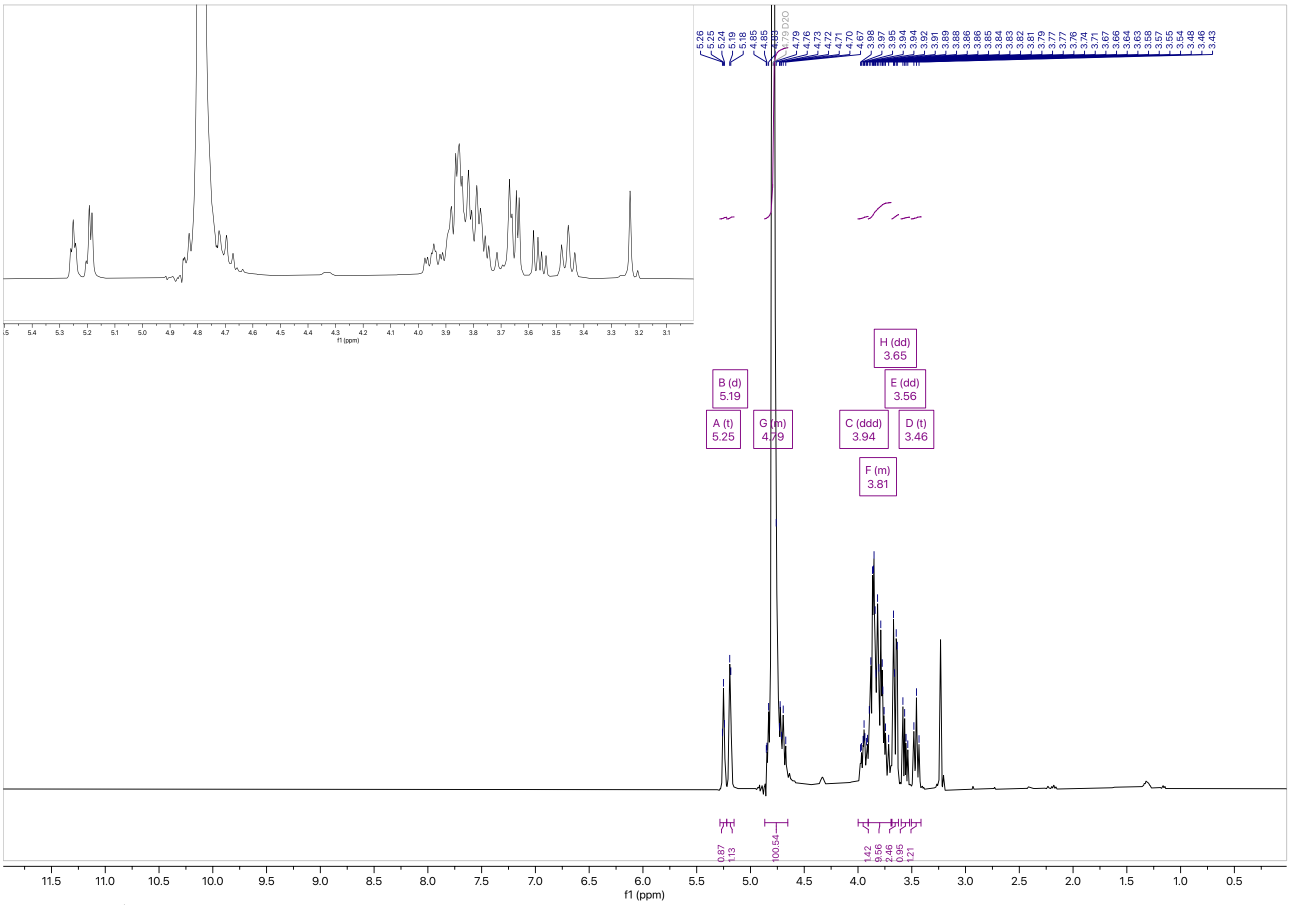

Fig. S15. ^{1}H NMR 3-deoxy-3-fluoro-trehalose (3) Inset shows the peaks zoomed in.

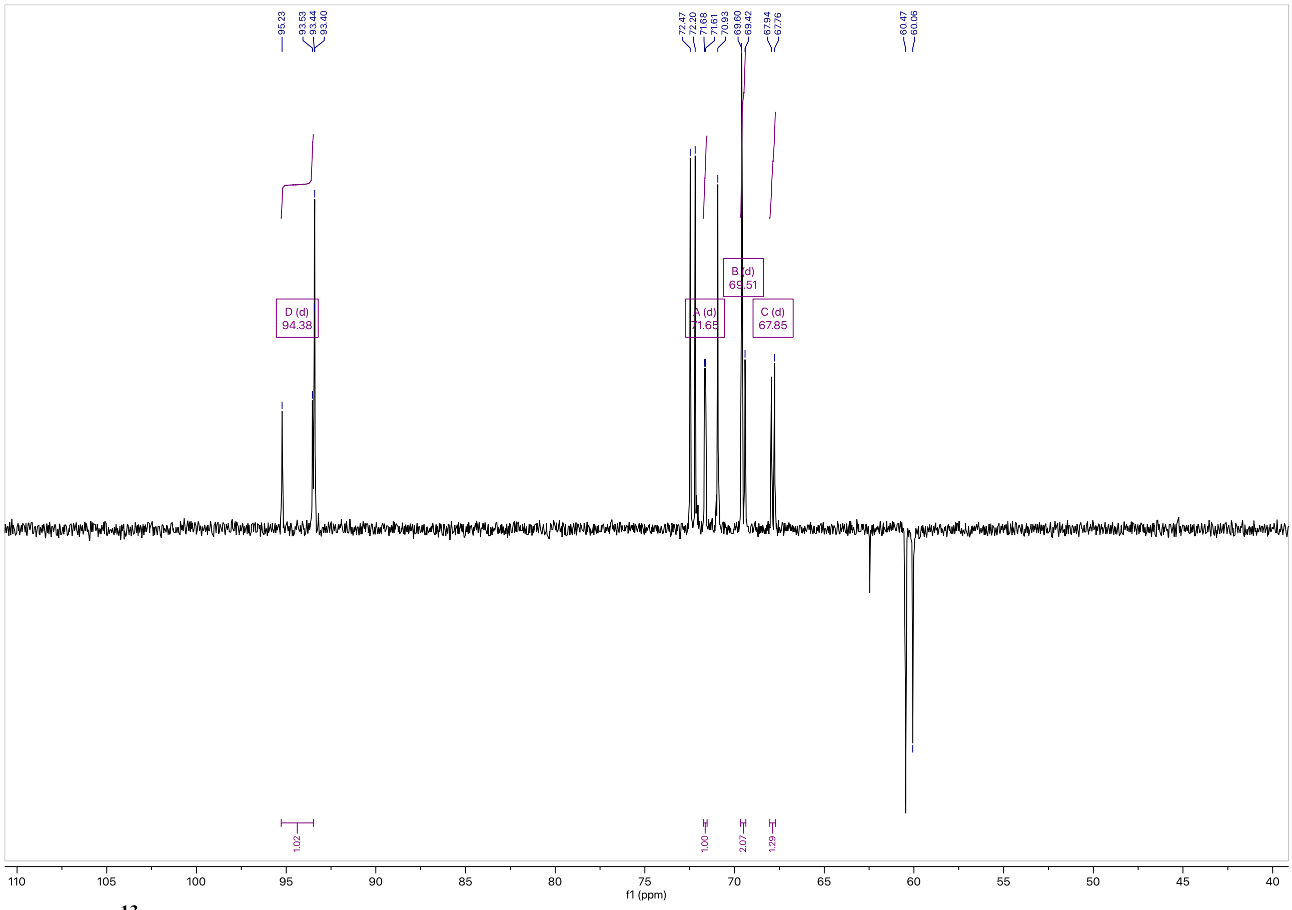

Fig. S16. ^{13}C NMR 3-deoxy-3-fluoro-trehalose (3)

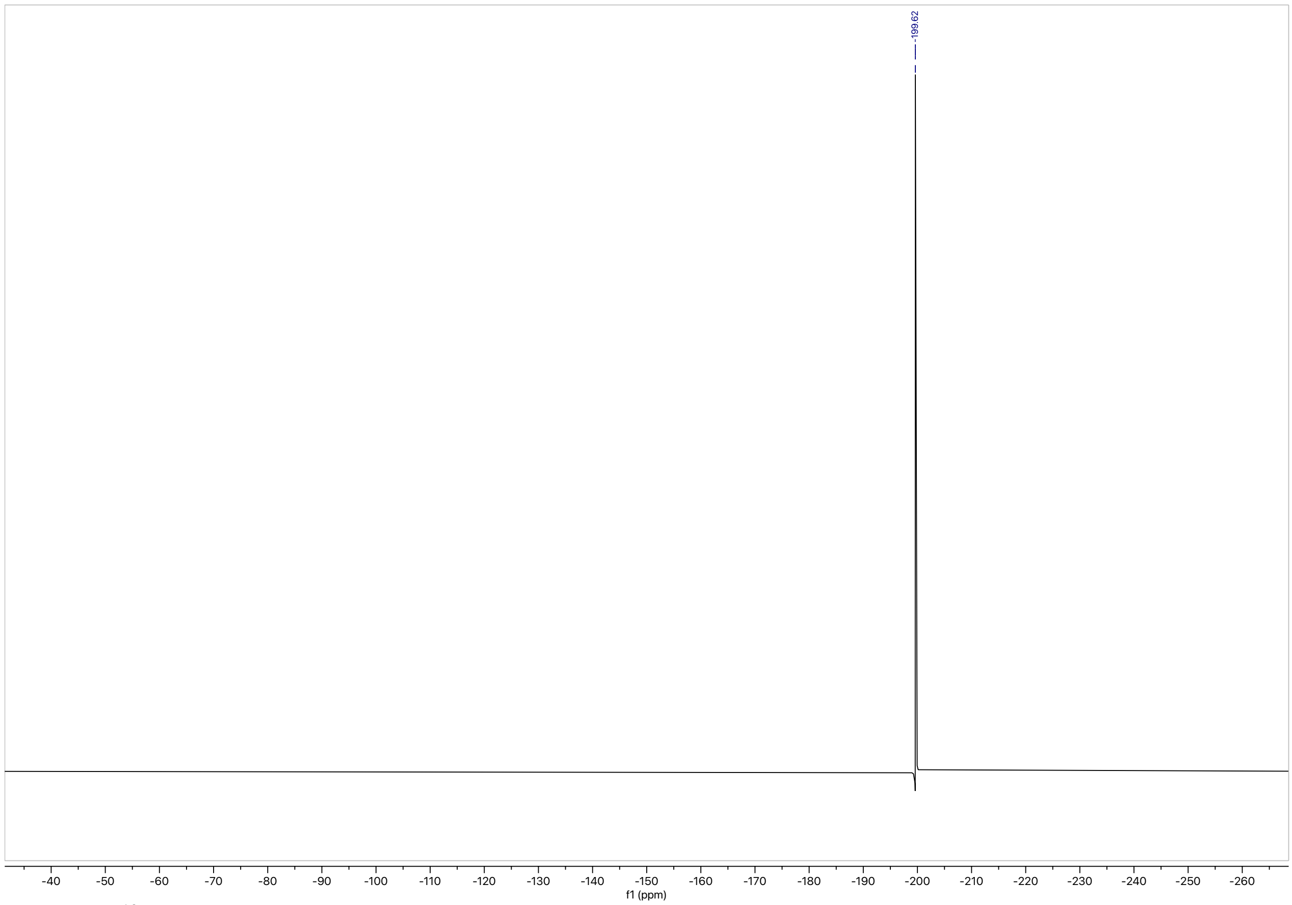

Fig. S17. ^{19}F NMR 3-deoxy-3-fluoro-trehalose (3)

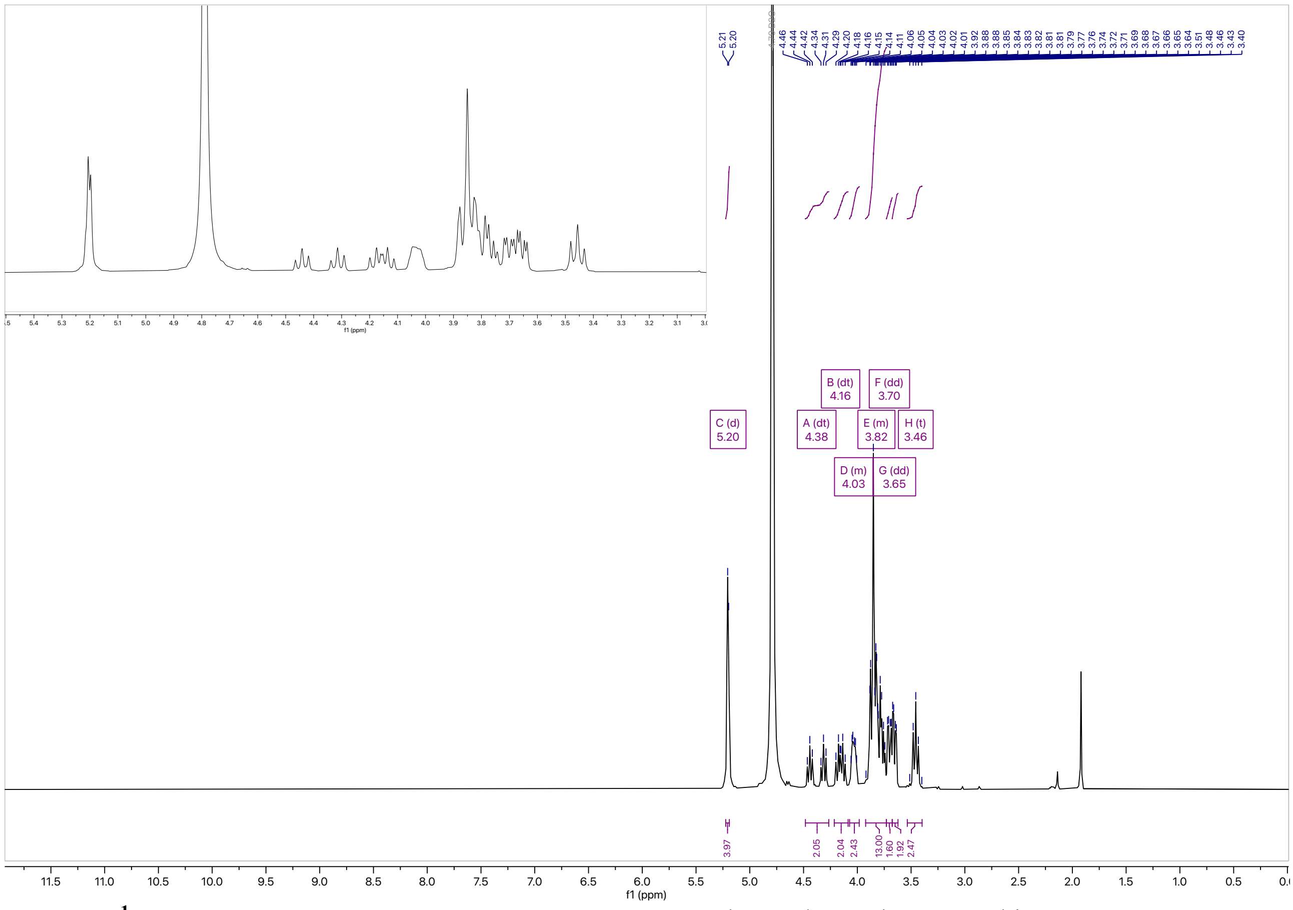

Fig. S18. ^{1}H NMR of 4-deoxy-4-fluoro-trehalose (4) Inset shows the peaks zoomed in.

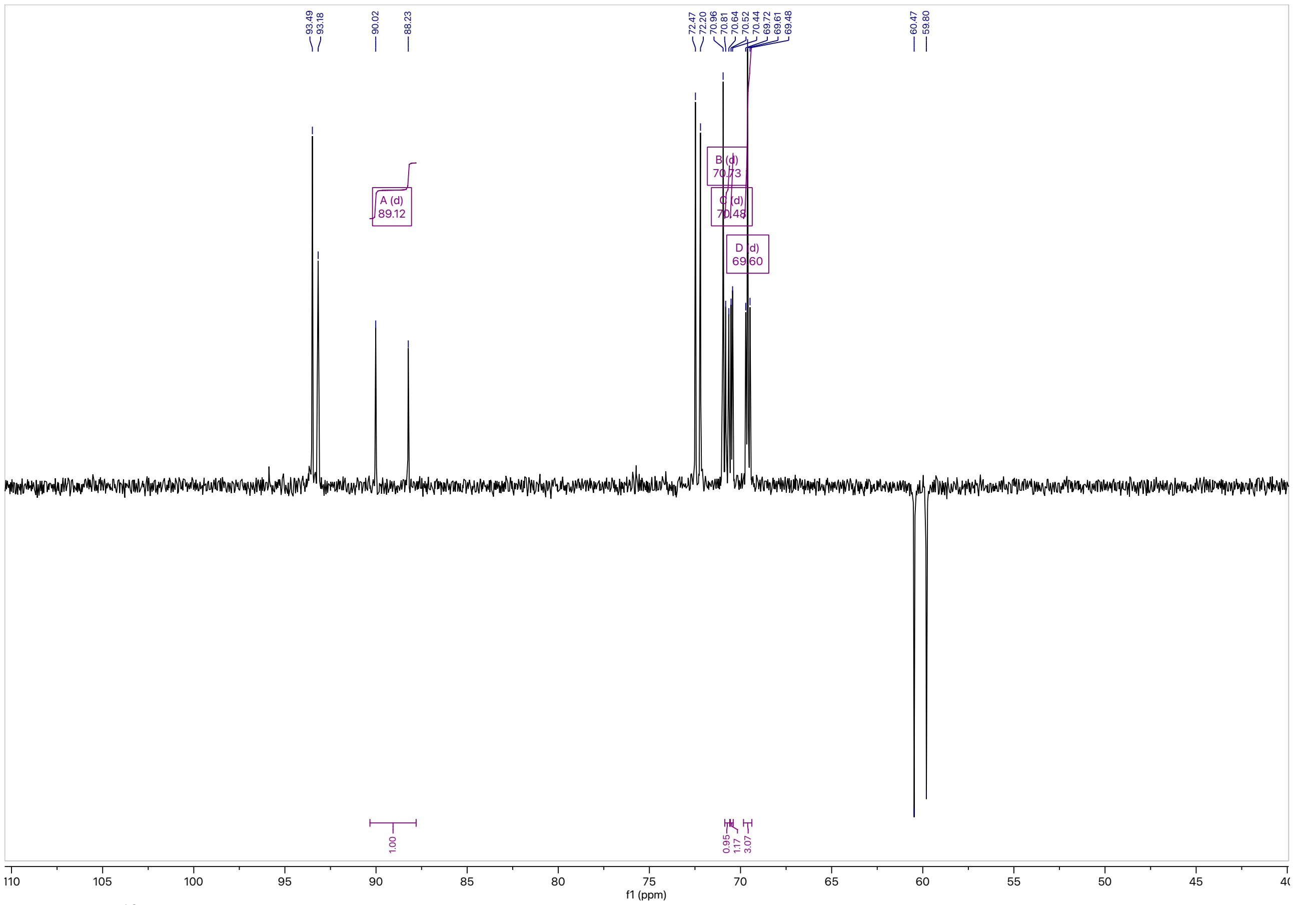

Fig. S19. ^{13}C NMR of 4-deoxy-4-fluoro-trehalose (4)

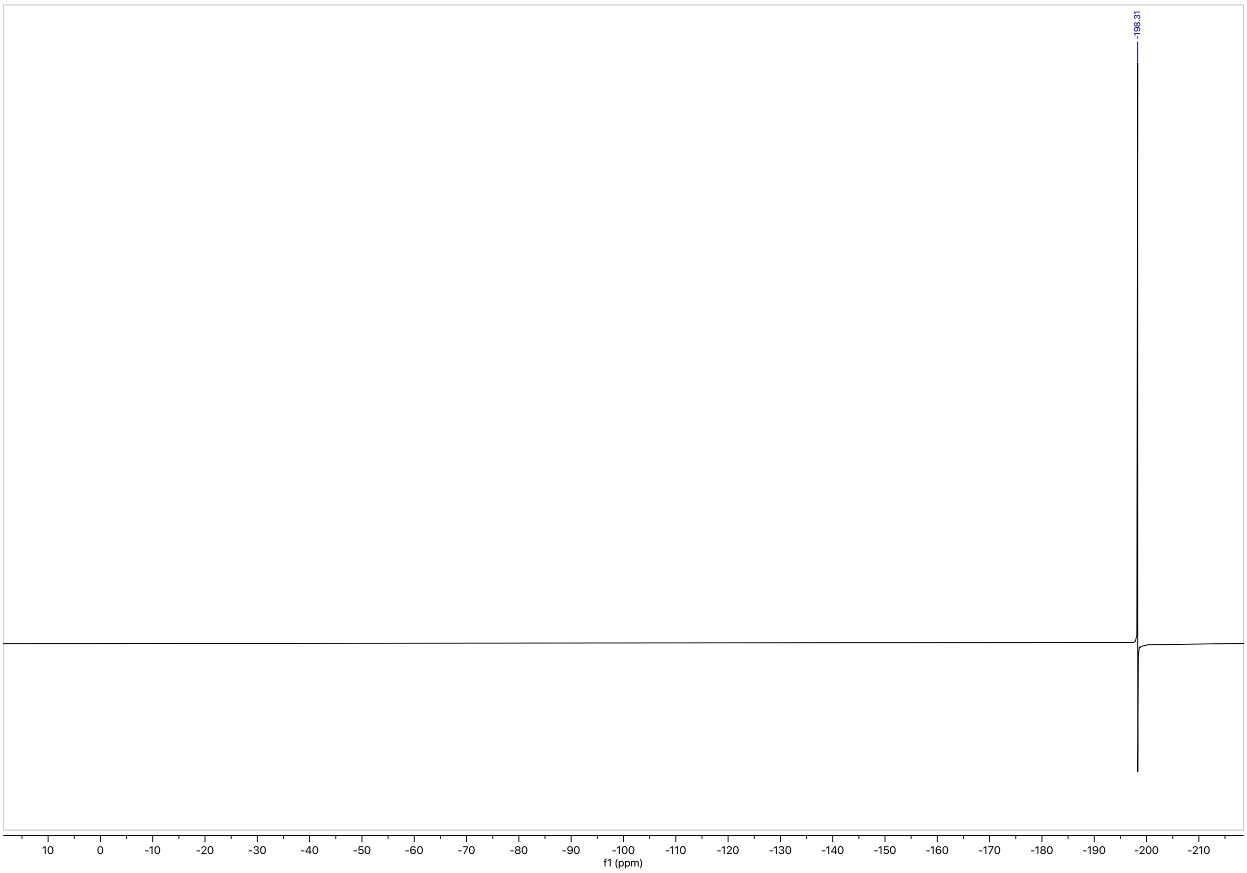

Fig. S20. ^{19}F NMR of 4-deoxy-4-fluoro-trehalose (4)

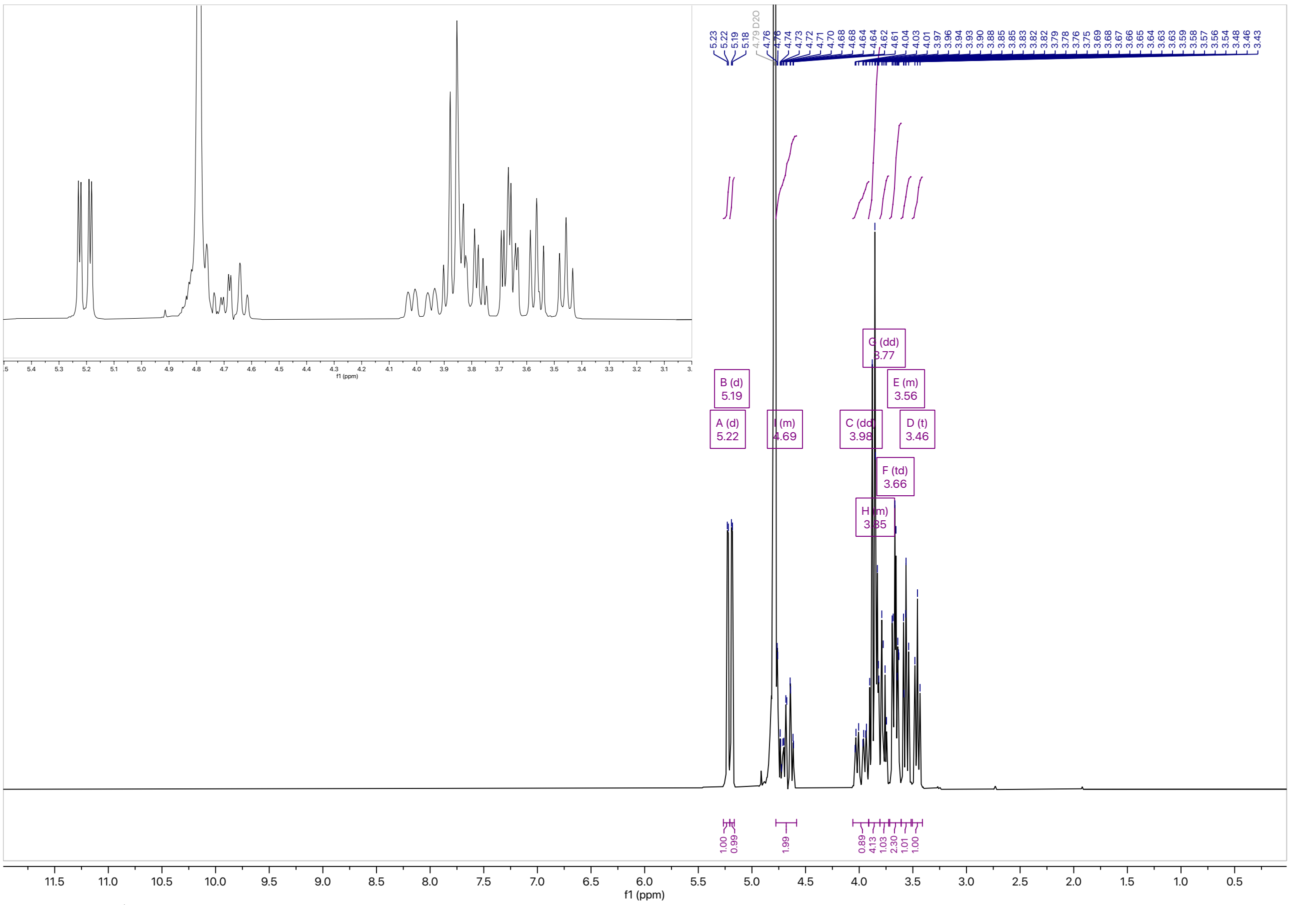

Fig. S21. ^{1}H NMR of 6-deoxy-6-fluoro-trehalose (5) Inset shows the peaks zoomed in.

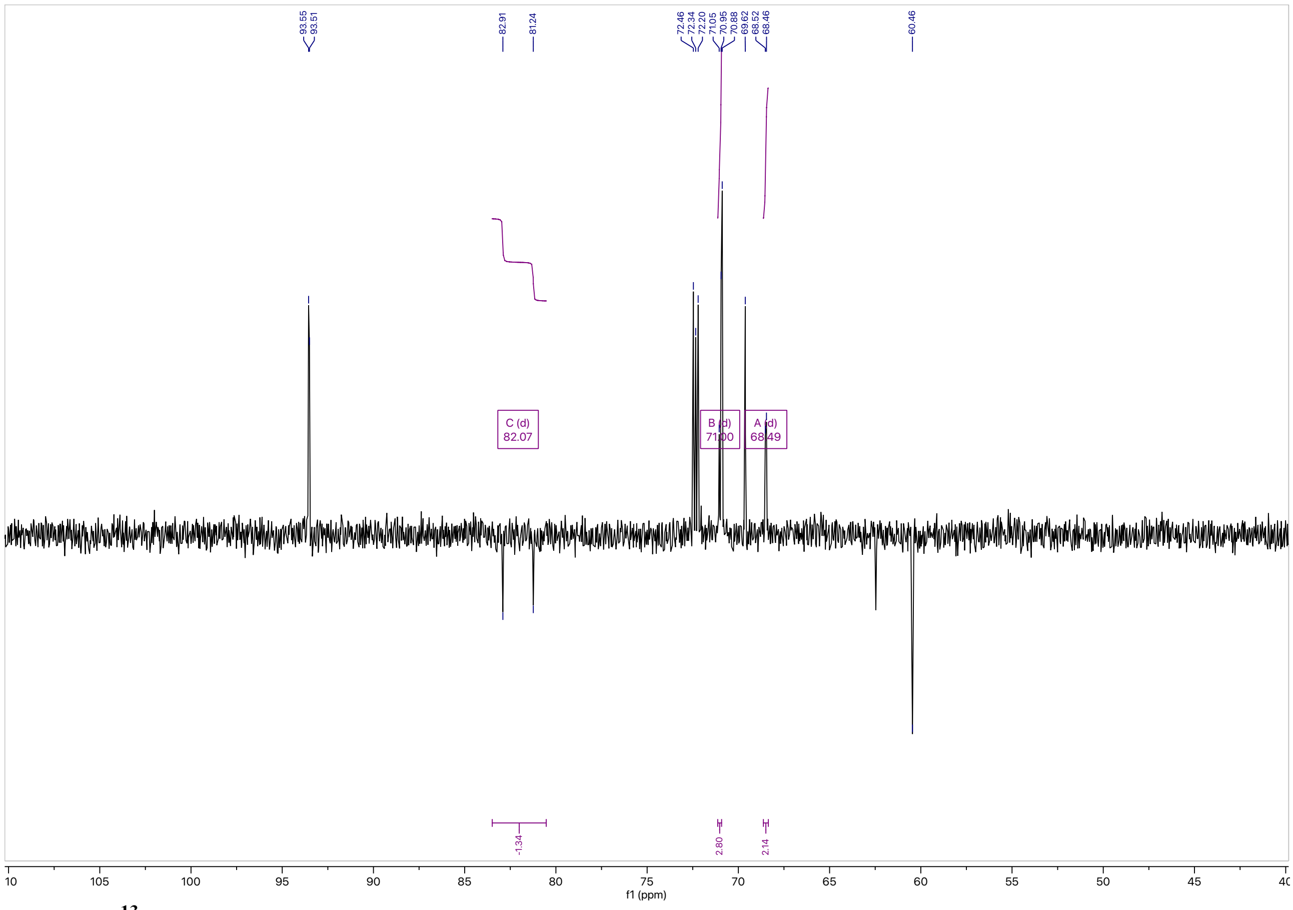

Fig. S22. ^{13}C NMR of 6-deoxy-6-fluoro-trehalose (5)

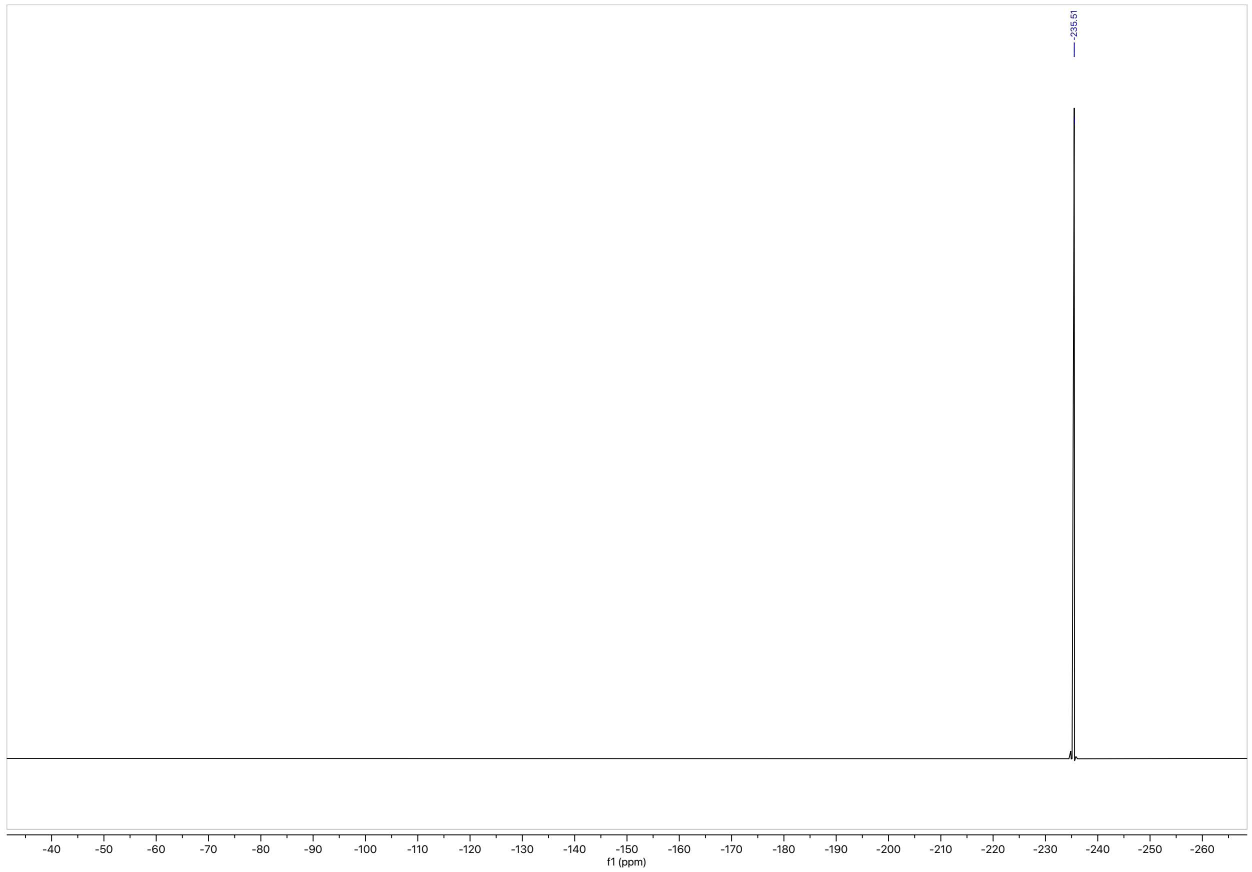

Fig. S23. ^{19}F NMR of 6-deoxy-6-fluoro-trehalose (5)

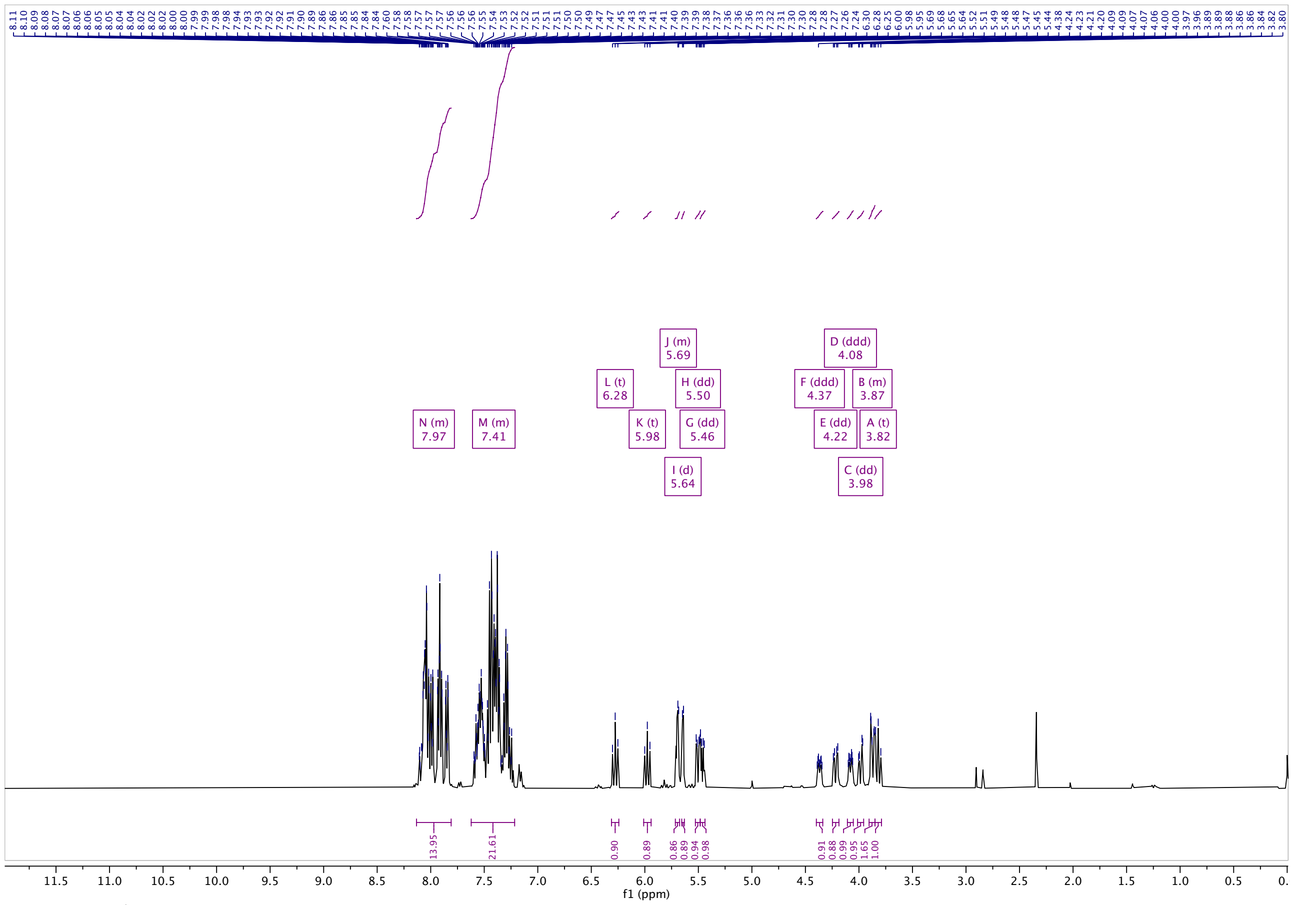

Fig. S24. ^{1}**H NMR of 2,3,6,2′,3′,4′,6’,-hepta-*O*-benzoyl-α,α’-D-trehalose (6)**

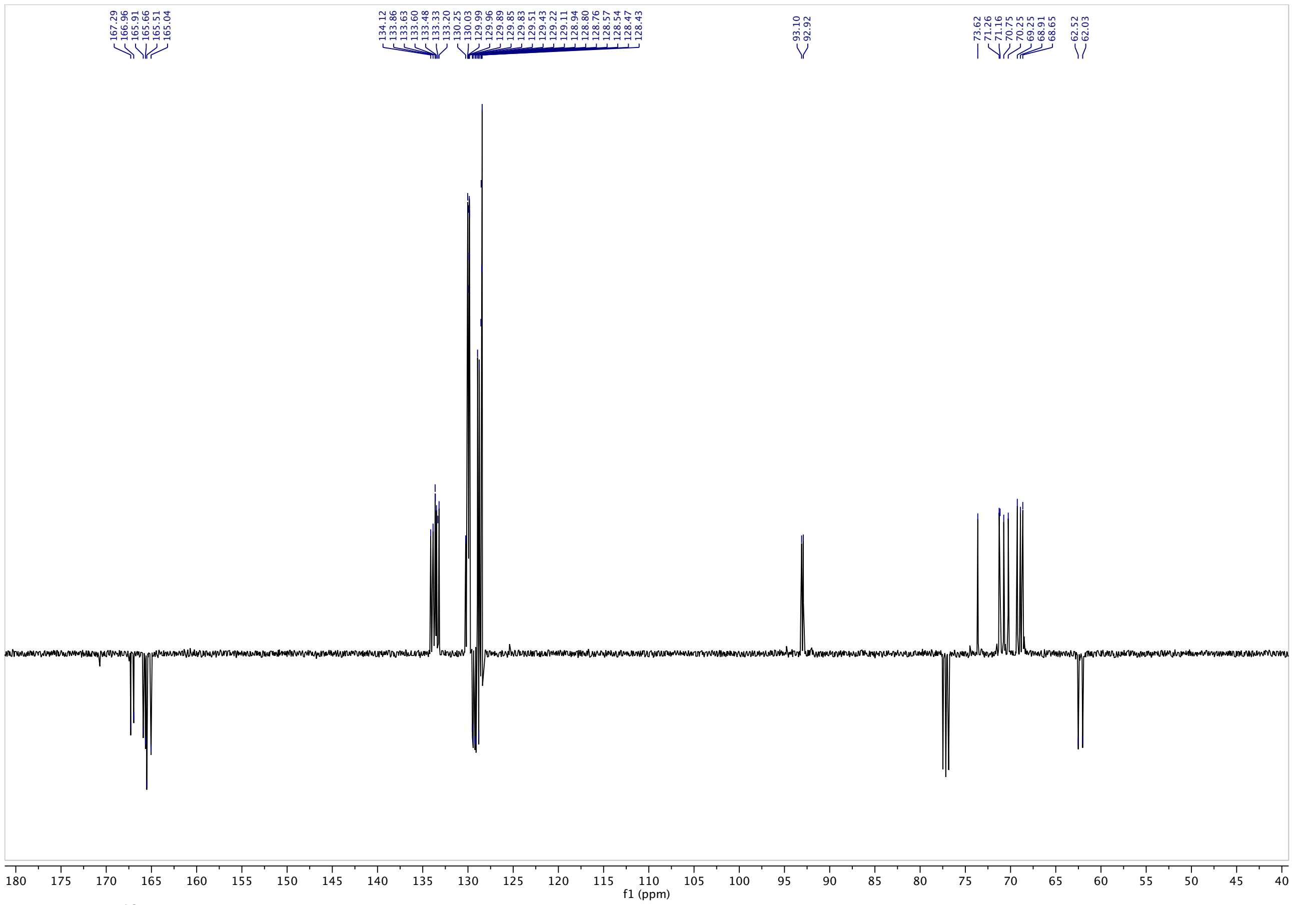

Fig. S25. ^{13}**C NMR of 2,3,6,2′,3′,4′,6’,-hepta-*O*-benzoyl-α,α’-D-trehalose (6)**

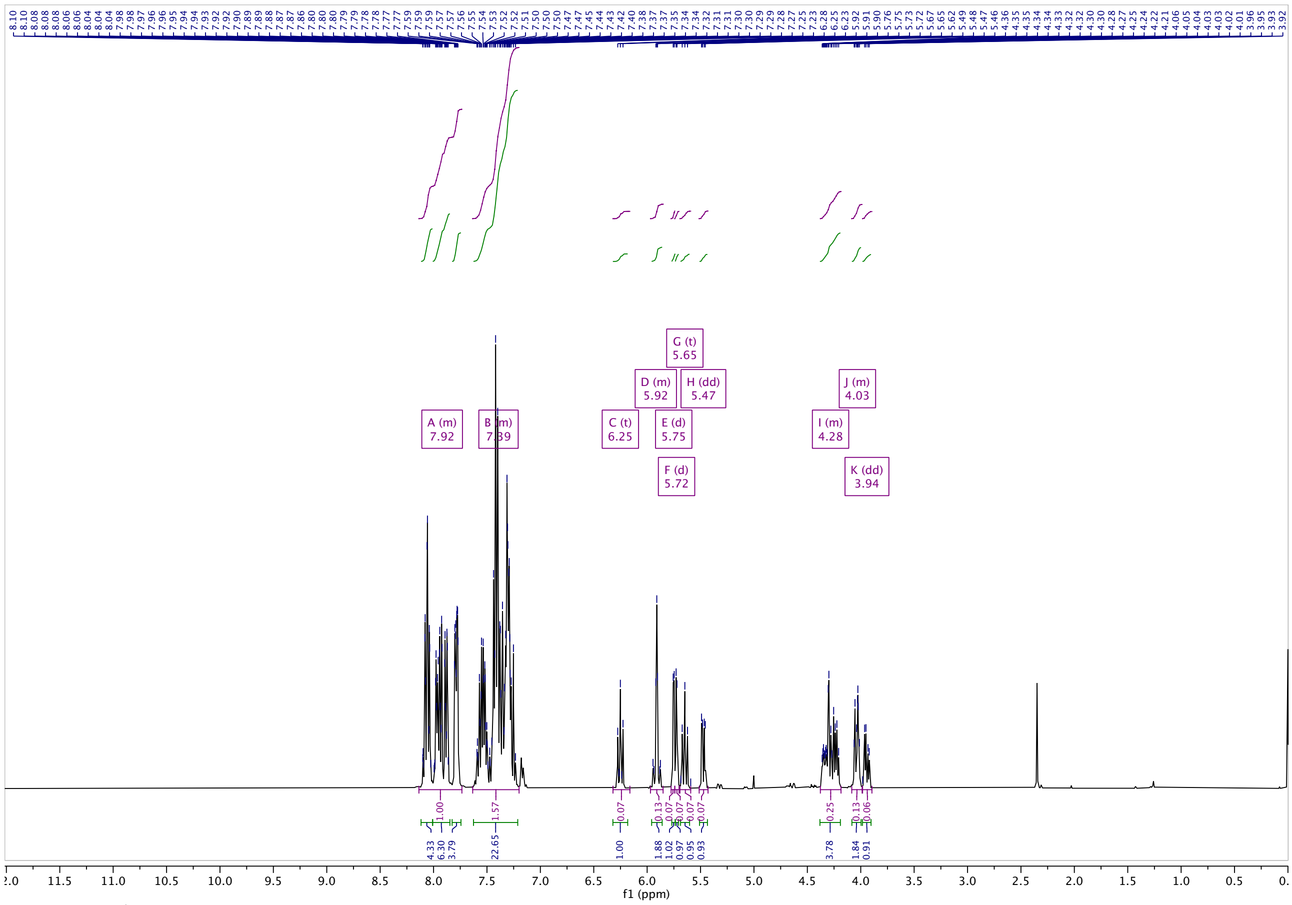

Fig. S26. ^{1}**H NMR of 2,3,6,-tri-*O*-benzoyl-α-D-galactopyranosyl-(1→1)-2′,3′,4′,6′,-tetra-*O*-benzoyl-α-D-glucopyranoside (8)**

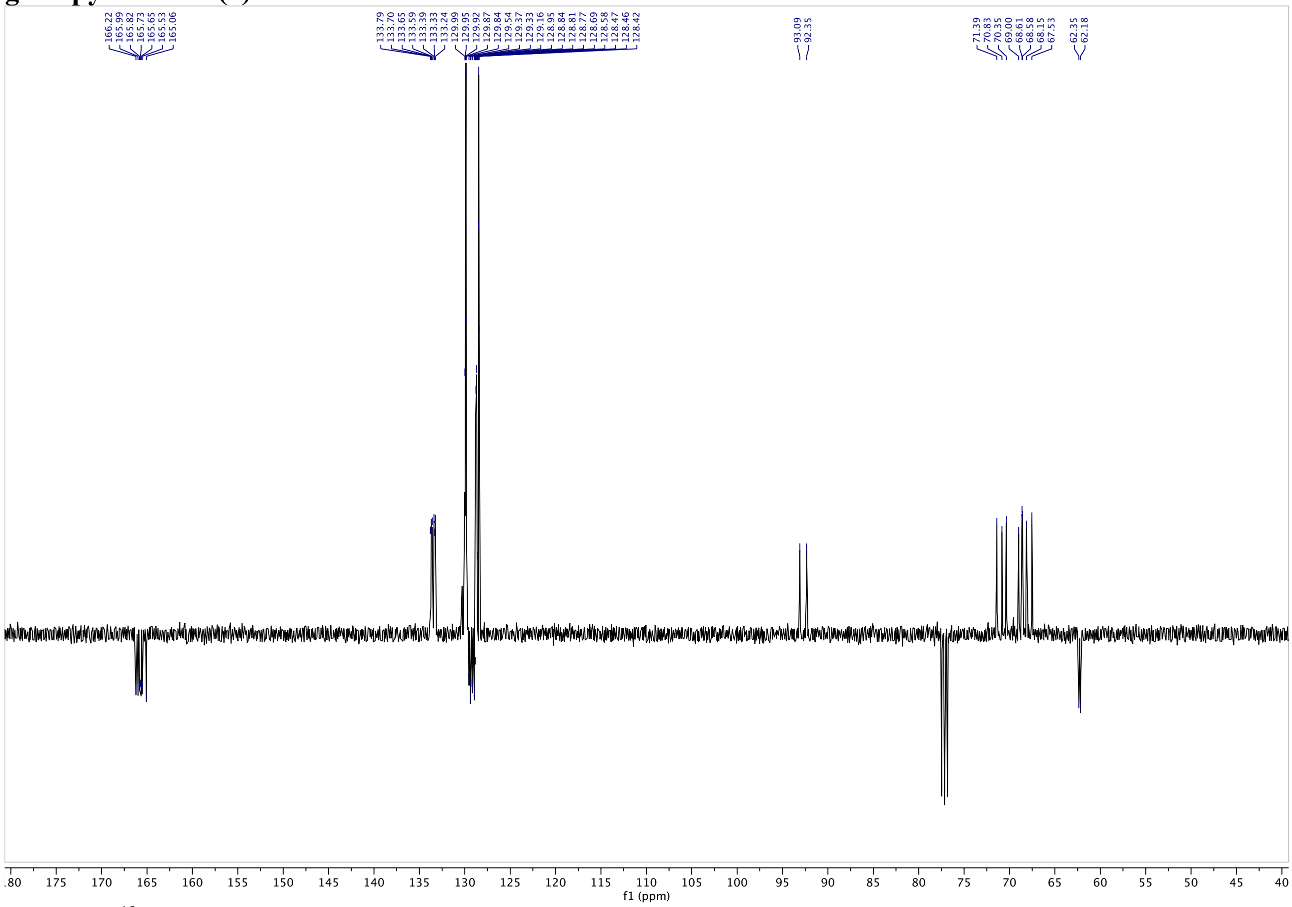

Fig. S27. ^{13}**C NMR of 2,3,6,-tri-*O*-benzoyl-α-D-galactopyranosyl-(1→1)-2′,3′,4′,6′,-tetra-*O*-benzoyl-α-D-glucopyranoside (8)**

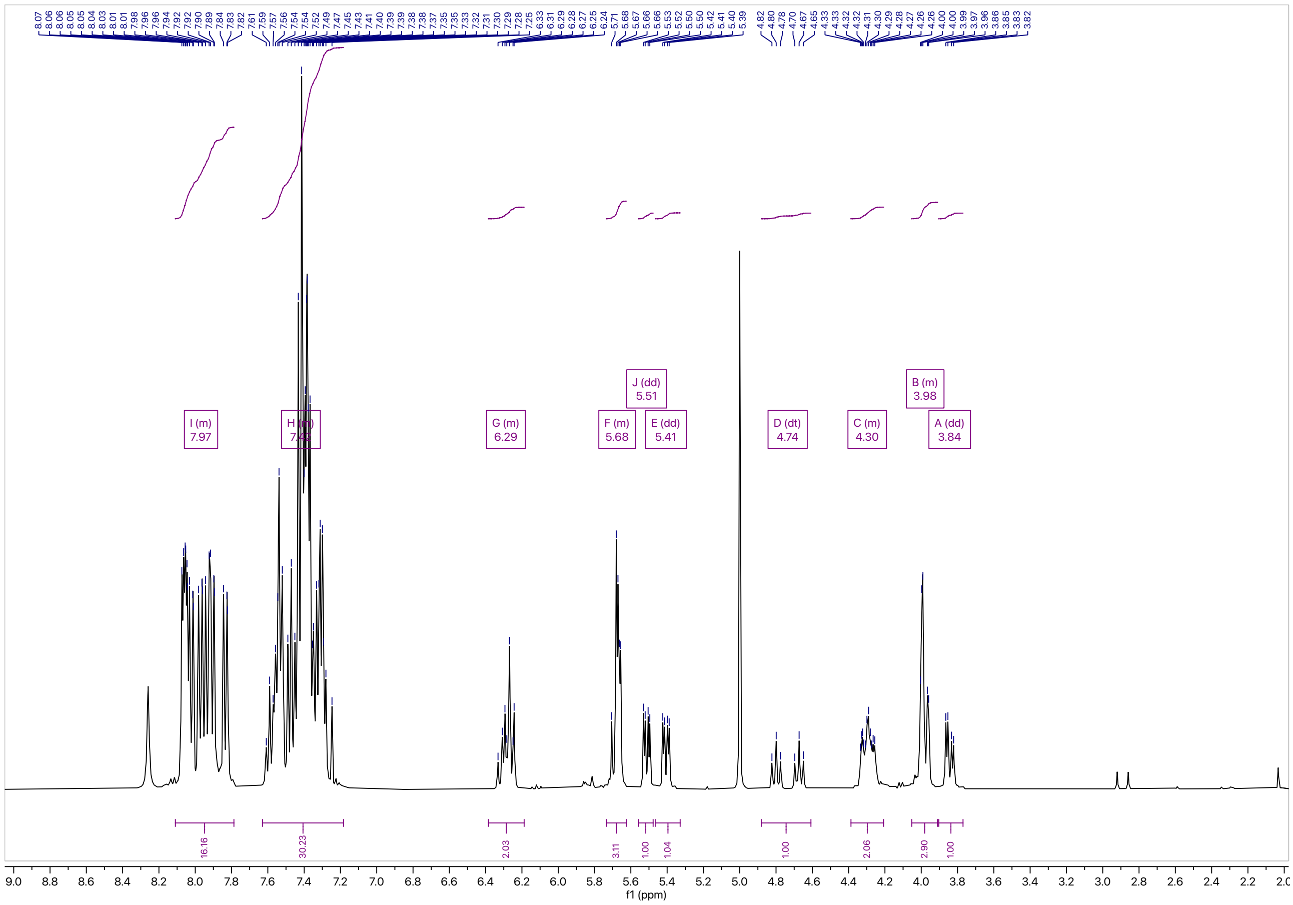

Fig. S28. ^{1}**H NMR of 4-fluoro-2,3,6,-tri-*O*-benzoyl-α-D-galactopyranosyl-(1→1)-2′,3′,4′,6′,-tetra-*O*-benzoyl-α-D-glucopyranoside (9)**

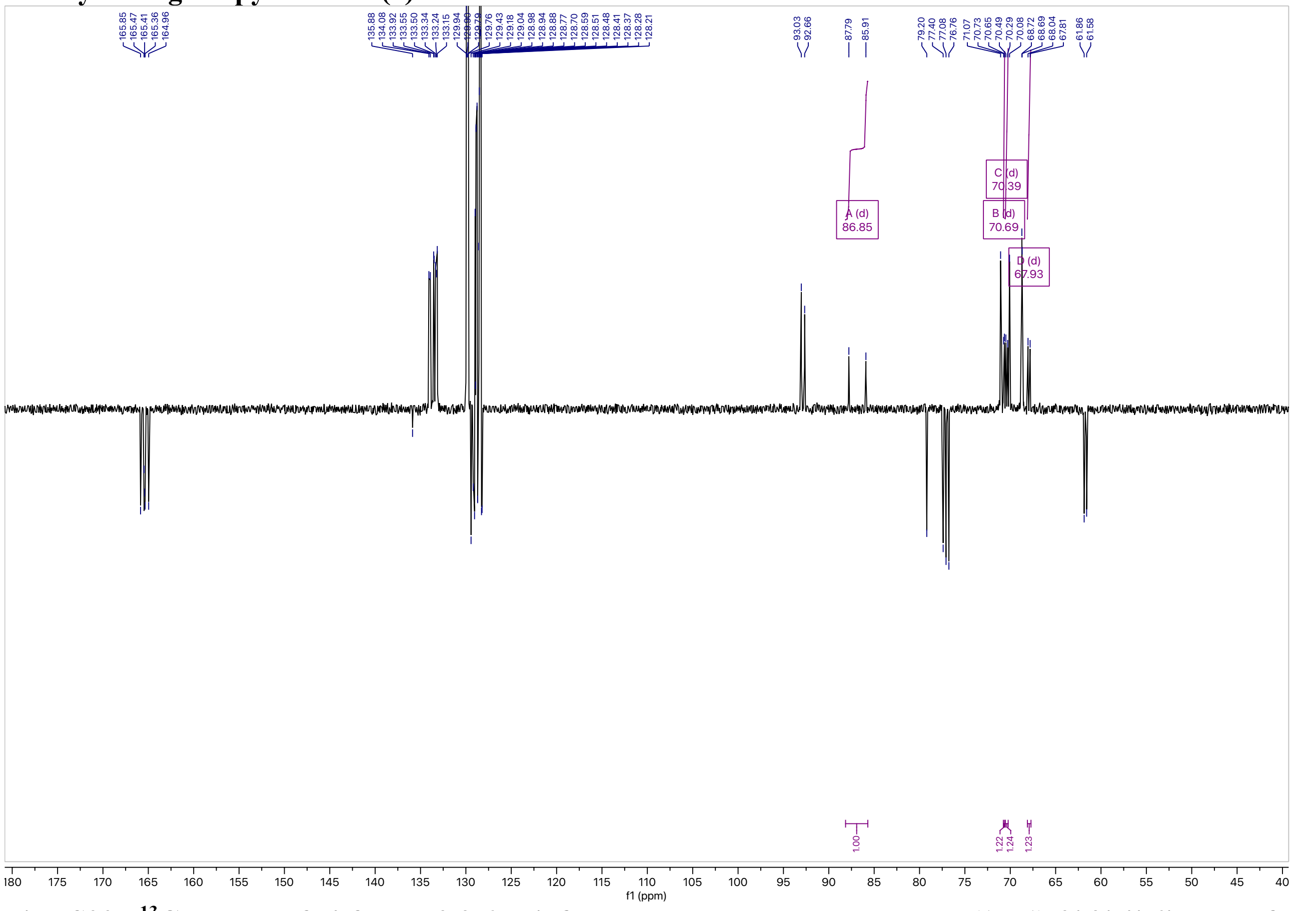

Fig. S29. ^{13}**C NMR of 4-fluoro-2,3,6,-tri-*O*-benzoyl-α-D-galactopyranosyl-(1→1)-2′,3′,4′,6′,-tetra-*O*-benzoyl-α-D-glucopyranoside (9)**

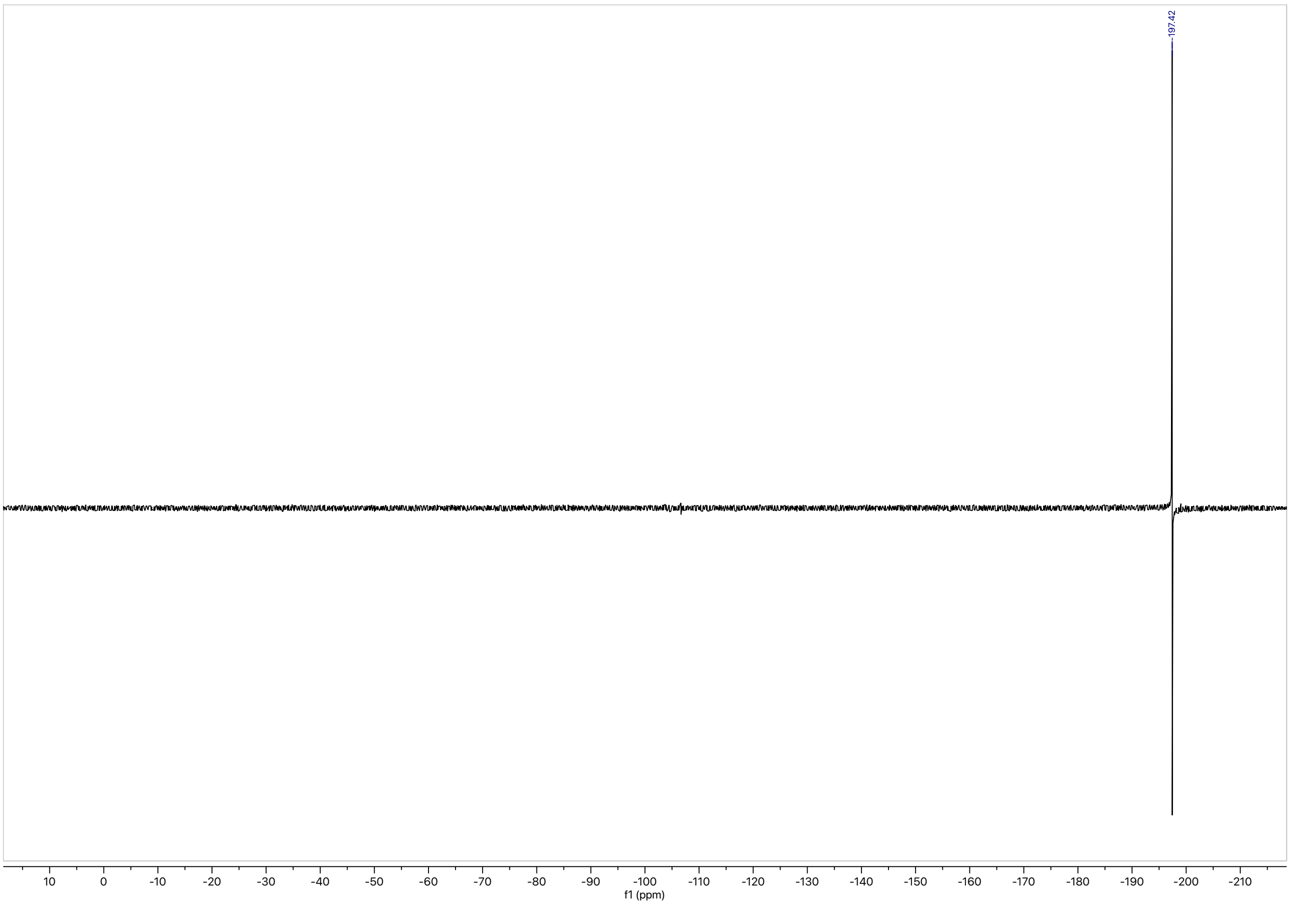

Fig. S30. ^{19}F NMR of 4-fluoro-2,3,6,-tri-*O*-benzoyl-α-D-galactopyranosyl-(1→1)-2′,3′,4′,6′,-tetra-*O*-benzoyl-α-D-glucopyranoside (9)

References

1. B. L. Urbanek, D. C. Wing, K. S. Haislop, C. J. Hamel, R. Kalscheuer, P. J. Woodruff and B. M. Swarts, Chemoenzymatic synthesis of trehalose analogues: rapid access to chemical probes for investigating mycobacteria, *ChemBioChem*, 2014, **15**, 2066-2070.
2. S. R. Rundell, Z. L. Wagar, L. M. Meints, C. D. Olson, M. K. O'Neill, B. F. Piligian, A. W. Poston, R. J. Hood, P. J. Woodruff and B. M. Swarts, Deoxyfluoro-d-trehalose (FDTre) analogues as potential PET probes for imaging mycobacterial infection, *Org Biomol Chem*, 2016, **14**, 8598-8609.
3. C. M. Furze, I. Delso, E. Casal, C. S. Guy, C. Seddon, C. M. Brown, H. L. Parker, A. Radhakrishnan, R. Pacheco-Gomez, P. J. Stansfeld, J. Angulo, A. D. Cameron and E. Fullam, Structural basis of trehalose recognition by the mycobacterial LpqY-SugABC transporter, *J Biol Chem*, 2021, **296**, 100307.
4. B. Han, Y. Liu, S. W. Ginzinger and D. S. Wishart, SHIFTX2: significantly improved protein chemical shift prediction, *J Biomol NMR*, 2011, **50**, 43-57.
5. S. Monaco, L. E. Tailford, N. Juge and J. Angulo, Differential Epitope Mapping by STD NMR Spectroscopy To Reveal the Nature of Protein-Ligand Contacts, *Angew Chem Int Ed Engl*, 2017, **56**, 15289-15293.
6. P. Andrio, A. Hospital, J. Conejero, L. Jorda, M. Del Pino, L. Codo, S. Soiland-Reyes, C. Goble, D. Lezzi, R. M. Badia, M. Orozco and J. L. Gelpi, BioExcel Building Blocks, a software library for interoperable biomolecular simulation workflows, *Sci Data*, 2019, **6**, 169.
7. J. A. Maier, C. Martinez, K. Kasavajhala, L. Wickstrom, K. E. Hauser and C. Simmerling, ff14SB: Improving the Accuracy of Protein Side Chain and Backbone Parameters from ff99SB, *J Chem Theory Comput*, 2015, **11**, 3696-3713.
8. J. Wang, R. M. Wolf, J. W. Caldwell, P. A. Kollman and D. A. Case, Development and testing of a general amber force field, *J Comput Chem*, 2004, **25**, 1157-1174.
9. W. L. Jorgensen, J. Chandrasekhar, J. D. Madura, R. W. Impey and M. L. Klein, Comparison of simple potential functions for simulating liquid water, *J. Chem. Phys.*, 1983, **79**, 926-935.
10. J. A. Izaguirre, D. P. Catarello, J. M. Wozniak and R. D. Skeel, Langevin stabilization of molecular dynamics, *J. Chem. Phys.*, 2001, **114**, 2090-2098.
11. K. B. D. M. Y. and P. A. K. D.A. Case, I.Y. Ben-Shalom, S.R. Brozell, D.S. Cerutti, T.E. Cheatham, III, V.W.D. Cruzeiro, T.A. Darden, R.E. Duke, G. Giambasu, M.K. Gilson, H. Gohlke, A.W. Goetz, R Harris, S. Izadi, S.A. Izmailov, K. Kasavajhala, A. Kovalenko, R. Krasny, T, AMBER 2020, *University of California, San Francisco, CA, USA, 2020*, 2020.
12. D. M. York, T. A. Darden and L. G. Pedersen, The effect of long-range electrostatic interactions in simulations of macromolecular crystals: A comparison of the Ewald and truncated list methods, *J. Chem. Phys.* , 1993, **99**, 8345-8348.
13. H. G. Petersen, Accuracy and efficiency of the particle mesh Ewald method, *J. Chem. Phys.* , 1995, **103**, 3668-3679.
14. J.-P. Ryckaert, G. Ciccotti and H. J. C. Berendsen, Numerical integration of the cartesian equations of motion of a system with constraints: molecular dynamics of n-alkanes, *J. Chem. Phys.*, 1977, **23**, 327-341.
15. S. Miyamoto and P. A. Kollman, Settle: An analytical version of the SHAKE and RATTLE algorithm for rigid water models, *J. Comput. Chem.*, 1992, **13**, 952-962.
